# Psychosocial supports for staff in maternity hospitals and units following adverse events: a mapping study in the Republic of Ireland

**DOI:** 10.1186/s12913-026-14465-7

**Published:** 2026-03-30

**Authors:** Marita Hennessy, Keelin O’Donoghue

**Affiliations:** 1 Pregnancy Loss Research Group, Department of Obstetrics and Gynaecology, University College Cork, Cork, Ireland; 2 INFANT Research Centre, University College Cork, Cork, Ireland

**Keywords:** Adverse events, Maternity care, Midwifery, Obstetrics, Psychosocial intervention, Stress, Vicarious trauma

## Abstract

**Background:**

Maternity staff commonly experience adverse events within their roles. Such events can have a profound impact, with a range of psychological impacts reported. Many maternity staff, however, report receiving inadequate support. The aim of this study is to establish the extent and nature of the provision of psychosocial supports for staff following adverse events in maternity hospitals and units in the Republic of Ireland.

**Methods:**

We administered a purposefully designed survey electronically to identified personnel in each of the 19 maternity hospitals and units from December 2023 to February 2024. The survey comprised primarily open-ended questions. Regarding staff supports following adverse events, respondents were asked whether they provided any of 13 pre-listed supports and they could add up to three additional supports. For each support provided, they were asked to detail its format, eligibility and access criteria, costs, evaluation, perceived uptake and impact, and any adaptations made over time. We analysed data descriptively, coding open-ended responses into explicit categories generated from the data and reporting frequencies, as per responses to closed questions.

**Results:**

We received completed responses on the staff survey from 18 of 19 maternity hospitals and units. All but one site (*n* = 17) reported the provision of at least one form of psychosocial support following adverse events, with numbers ranging from 2 to 10 (mode = 6). The most frequently reported supports offered were Employee Assistance Programme (*n* = 16, 89%); Occupational Health (*n* = 15, 83%); Clinical Supervision (*n* = 10, 56%); After Action Review (*n* = 9, 50%). Varied information was provided about each of the supports within and across sites. While staff listed supports that were available in their hospital, they were unable to provide much detail regarding these, particularly regarding their evaluation, uptake and impact. There was also confusion between the different types of supports, especially relating to various forms of debriefing.

**Conclusions:**

This study maps the provision of psychological supports for staff in maternity hospitals and units in the Republic of Ireland following adverse events. Further research is needed to better understand what the optimal staff supports are, and what factors influence their implementation, to enhance uptake and impacts.

**Supplementary Information:**

The online version contains supplementary material available at 10.1186/s12913-026-14465-7.

## Background

Maternity staff commonly experience high risk situations or adverse events – including traumatic births, the death of a baby before, during or after birth, and maternal death – within their roles. Such events can have a profound impact on the staff involved [1–13]. In their systematic review of the perceived impact of birth trauma witnessed by maternity health professionals, Uddin et al. found that 45–97% of staff within included studies had witnessed a traumatic birth event, with the prevalence of secondary traumatic stress ranging from 13 to 39%, and 3–46% meeting diagnostic criteria for post-traumatic stress disorder (PTSD) [12].

Maternity staff who witness or are involved in adverse or traumatic maternity events report a range of psychological impacts including depression, anxiety, stress, PTSD, fear, guilt, sleep disorders, compassion fatigue and burnout [1, 9, 11, 12, 14]. Factors within the broader landscape – such as workplace culture and human resource procedures, how investigations of adverse events are conducted, negative media coverage and fear of litigation – can magnify negative impacts experienced [7, 9, 15]. Adverse events can also make maternity staff question their professional identify and whether they want to continue working in the area or not, with some leaving the profession entirely [1, 9, 12, 16]. Given the recruitment and retention concerns in maternity care, supporting staff surrounding adverse events, and their wellbeing more generally, is important [9]. While many maternity staff appear to be negatively affected by traumatic events, many report receiving inadequate support [8, 9], which can compound negative impacts on psychological wellbeing [7]. In a UK study, trainees in obstetrics and gynaecology who perceived less support after serious incidents reported engaging in defensive medical practices, and physical and/or mental health problems, slightly more than those who felt well supported [17].

Interventions to prevent or reduce occupational stress and burnout in healthcare staff show promise; however, gaps remain as to which interventions are most effective in specific populations, as well as how to combine individual and organisational solutions to maximise impacts [18, 19]. In their review of interventions for midwives and student midwives in work-related psychological distress, Pezaro and colleagues identified mindfulness interventions, work-based resilience workshops alongside mentoring programmes, and clinical supervision as having positive impacts though noted that some reported less favourable experiences and there were practical barriers to uptake for others [20]. While (to our knowledge) no review has specifically examined psychosocial support interventions for a broader range of maternity staff, some interventions targeting this specific population have been conducted. For example, in a pilot study, Wallbank [21] found that individual clinical supervision had a positive impact on reducing stress in doctors and midwives in maternity care, and maternity staff in a large hospital in Ireland have reported positive experiences of attending structured staff forums such as Schwartz Rounds [22, 23].

Audits of perinatal bereavement care in the 19 maternity hospitals and units (herein referred to as ‘maternity units’) across the Republic of Ireland, from 2017 to 2020, identified some staff supports in operation; details were limited, with the Health Service Executive (HSE)’s Employee Assistance Programme (EAP) the most commonly cited form of support [24]. Better understanding of what supports are provided in practice is needed to inform future implementation efforts, including opportunities to standardise and scale up interventions nationally. The aim of this study therefore is to establish, for the 19 maternity units in the Republic of Ireland, the extent and nature of the provision of psychosocial supports for staff following adverse events, including pregnancy loss. We also sought to examine the uptake, evaluation and perceived impacts of such supports. For the purposes of this study we define an adverse event as ‘an incident which resulted in harm that may or may not be the result of an error’, in line with the national (HSE) Incident Management Framework [25]. Within the Framework, harms are categorised as negligible, minor, moderate, major, and extreme, with associated definitions/examples for each provided.

## Methods

### Study design

To achieve the stated aims and objectives we conducted a cross-sectional online survey to determine what supports are provided and/or available for staff in maternity units in the Republic of Ireland. This was part of a wider national survey which also sought to establish the extent and nature of the provision of staff education and training opportunities on perinatal bereavement care and implementation influences, the findings of which are reported elsewhere [26].

### Ethical approval

The Clinical Research Ethics Committee of the Cork Teaching Hospitals granted ethical approval for this study (ref ECM4(u)24/10/2023). Respondents provided informed consent to undertake the survey, further to reading the participant information sheet which was embedded within the online survey platform [see survey in Additional File 1]. They were assured that participation was voluntary, that completion and submission of the survey responses indicated continued consent and that they could withdraw from the study at any point before survey submission by closing their web browser. We also highlighted that individual participants and/or their hospitals or units would not be identified in any reporting of the study findings and that the aim of the study was to establish what is currently provided nationally, not to evaluate practices within individual hospitals or units.

### Study context

The need for appropriate supports for staff surrounding adverse or traumatic events is highlighted within a range of policy documents in the Republic of Ireland. The National Standards for Bereavement Care following Pregnancy Loss and Perinatal Death in Ireland reflect the need for peer support and professional support systems, including self-care, peer support, debriefing, feedback and formal/informal support [27]. One of the standards specifically focuses on the need for hospital management to recognise the importance of providing debriefing for staff involved with trauma or sudden deaths and put formal and informal systems in place [27]. Another emphasises the importance of formal and informal evaluations of staff supports [27]. A review of the implementation of the Standards noted the need for the introduction of Schwartz Rounds or other support programmes [24].

Staff support is a key principle of open disclosure, as laid out within the National Open Disclosure Framework [28] and the HSE’s Open Disclosure Policy [29]. These acknowledge the importance of identifying and supporting the staff involved in and/or affected by the patient safety incident in both the immediate aftermath and on an on-going basis for as long as necessary. After Action Review (a structured review process focused on learning for improvement [30]) forms part of the National Incident Management Framework in the Irish health services, in addition to other review and/or support measures [25].

There are 19 publicly-funded maternity units operating the Republic of Ireland. In 2022, there were 54,539 births across all units, with four units accounting for over 6,000 births each [31] [see details in Additional File 2]. Maternity care is provided through three different pathways, all within a multi-disciplinary framework: supported care (midwifery-led), assisted care (obstetrics-led, delivered by obstetricians and midwives) and specialised care (obstetrics-led, delivered by obstetricians and midwives) [32]. All of the 19 publicly-funded maternity units are subject to policies of the HSE, including open disclosure, and all have access to the HSE’s EAP.

### Survey design

Participants were invited to complete a purposefully designed survey. The survey instrument was developed around the audit tool for the National Standards for Bereavement Care following Pregnancy Loss and Perinatal Death, supplemented with questions adapted from behavioural/implementation science frameworks such as the RE-AIM (Reach, Effectiveness-Adoption, Implementation, Maintenance) and TIDIER (Template for intervention description and replication) frameworks [33, 34] and following methodological guidance for qualitative surveys [35]. Defined staff supports were selected for inclusion by the authors based on knowledge of what staff supports were potentially available to staff in line with national guidance [30, 36] and the aforementioned audits. The survey comprised primarily open-ended questions, divided into four sections: (1) About you / your maternity hospital or unit, (2) Staff education and training on bereavement care, (3) Staff supports surrounding adverse events, (4) Any other comments. This study reports on sections 1, 3 and 4 of the full (overall) survey which can be viewed in Additional File 1.

In the staff supports section, respondents were asked to state whether they provided any of the pre-defined supports which were listed (13 in total; see Table 1) and were also given the opportunity to add details of up to three additional supports. For each support provided, respondents were asked to provide details across a range of domains, including format of the support, eligibility or access criteria, costs, uptake, if any evaluation was undertaken, perceived impact and any adaptations made to the support over time. In the ‘any other comments’ section, respondents were asked to note anything else they wished to tell the research team about staff supports following adverse events. Survey questions were discussed with members of the Pregnancy Loss Research Group (PLRG), National Women and Infants Health Programme, and the Advisory Group for the Implementation of the National Standards for Bereavement Care following Pregnancy Loss and Perinatal Death. We piloted the survey with members of the PLRG.

In the survey instructions, respondents were asked to provide as much information as they could for each question. The overall survey took approximately 45–60 min to complete, depending on the level of detail provided.

**Table 1:** Types of supports

Support type	Definition or guidance provided
After Action Reflection	*An event debriefing process which focuses on staff wellbeing and/or learning for improvement*
After Action Review	“A structured review process which seeks to rapidly identify and reinvest learning for improvement” [30]
Ballint Group	“Purposeful, regular meeting among clinicians, facilitated by trained leaders, who discuss the doctor-patient relationship and provide peer support” [37]
Clinical Supervision	“Professional relationship between a supervisor and a supervisee where the supervisor facilitates the practitioner in reflecting critically upon their practice” (One-to-One) [38]
Crisis Intervention (Other)	Note: Please do not include CISM or TRiM here; they are covered under separate categories
Critical Incident Debriefing	Note: Excluding CISM/TRiM
Critical Incident Stress Management (CISM) Response	An emergency mental health intervention, which is a form of psychological first aid [39]
Employee Assistance Programme (EAP): Staff Counselling (One-to-One)	*One of the service provided through EAP; usually a short-term service with staff initially offered up to six individual counselling sessions* [40]
Hospital Psychologist	Who can provide individual/group counselling and psychological support(s)
Occupational Health: HSE Staff	“*Provides an independent and confidential advisory service to employees and the employer on matters relating to the ‘effect of health on work’ and ‘work on health’*” [41]
Professional (External) Counselling Services (One-to-One)	*A safe and confidential space to talk to a trained professional – external to the organisation - about issues and concerns* [42]
Schwartz Rounds	Structured forum where all staff come together regularly to discuss the emotional and social aspects of working in healthcare [43]
Trauma Risk Management (TRiM)	A trauma-focused peer support system designed to help people who have experienced a traumatic, or potentially traumatic, event [44]

Note: Definitions in italics were not provided in the original survey as they are commonly understood supports/terms (i.e. EAP counselling, Occupational Health, Professional Counselling) and/or we did not want to limit participants’ understanding/definition of the support but rather further explore it in the survey (i.e. After Action Reflection)

### Participant recruitment and survey administration

The survey was administered electronically using Qualtrics, with the option available to complete it via virtual, telephone or in-person interview (i.e. interviewer-administered), if needed. Before participant recruitment commenced, the study sponsors–the National Women and Infants Health Programme–sent a letter (electronically) to each of the 19 maternity units advising them that the study was taking place. The research team then emailed the Clinical Leads for Pregnancy Loss and Directors of Midwifery or Nursing (DOM) within each of the 19 sites inviting them, or a representative, to take part in the study. The Clinical Midwife Specialists in Bereavement and Loss (CMS-BL) at each site were copied on correspondence to ensure that they were aware of the study also. The research team issued reminders to complete the survey via email, telephone or SMS. The survey was open to responses from 05 December 2023 to 05 February 2024.

### Data preparation and analysis

Survey data was downloaded from Qualtrics in Microsoft Excel. The lead researcher (MH) reviewed the dataset and prepared it for analysis. This included removing any identifying names and pseudo-anonymising the data. We also merged responses in cases where multiple responses were received from a maternity unit. We analysed data descriptively in MS Excel. We coded open-ended responses into explicit categories generated from the data and reported frequencies (as per responses to closed questions). We describe our analytical approach as quantitative content analysis; we did not use qualitative content analysis as we were reporting specific features of staff supports, not depth of insight or meaning attributed to them [45]. Limited information was provided regarding staff supports in Section 4 of the survey (question regarding any other comments) and therefore we did not include this in our analysis. Five out of the 18 sites provided some form of response, mainly noting the need for and importance of staff supports.

## Results

We received at least one response from each of the 19 maternity units in the Republic of Ireland to the overall survey, receiving 22 surveys in total. Respondents self-identified as CMS-BL (*n* = 14), A/DOM (*n* = 5), CMS-BL and ADOM (*n* = 1, joint response), Consultant Obstetrician/Gynaecologist (*n* = 1) and Midwifery Clinical Skills Facilitator (*n* = 1). It should be noted that, anecdotally, we are aware that some responses were prepared by a team of staff and submitted by one staff member on their behalf. While all 19 maternity units completed the education programme section of the survey, only 18 completed the section on staff supports (i.e. the focus of this study), giving a response rate of 18/19 (95%).

**Fig. 1:**
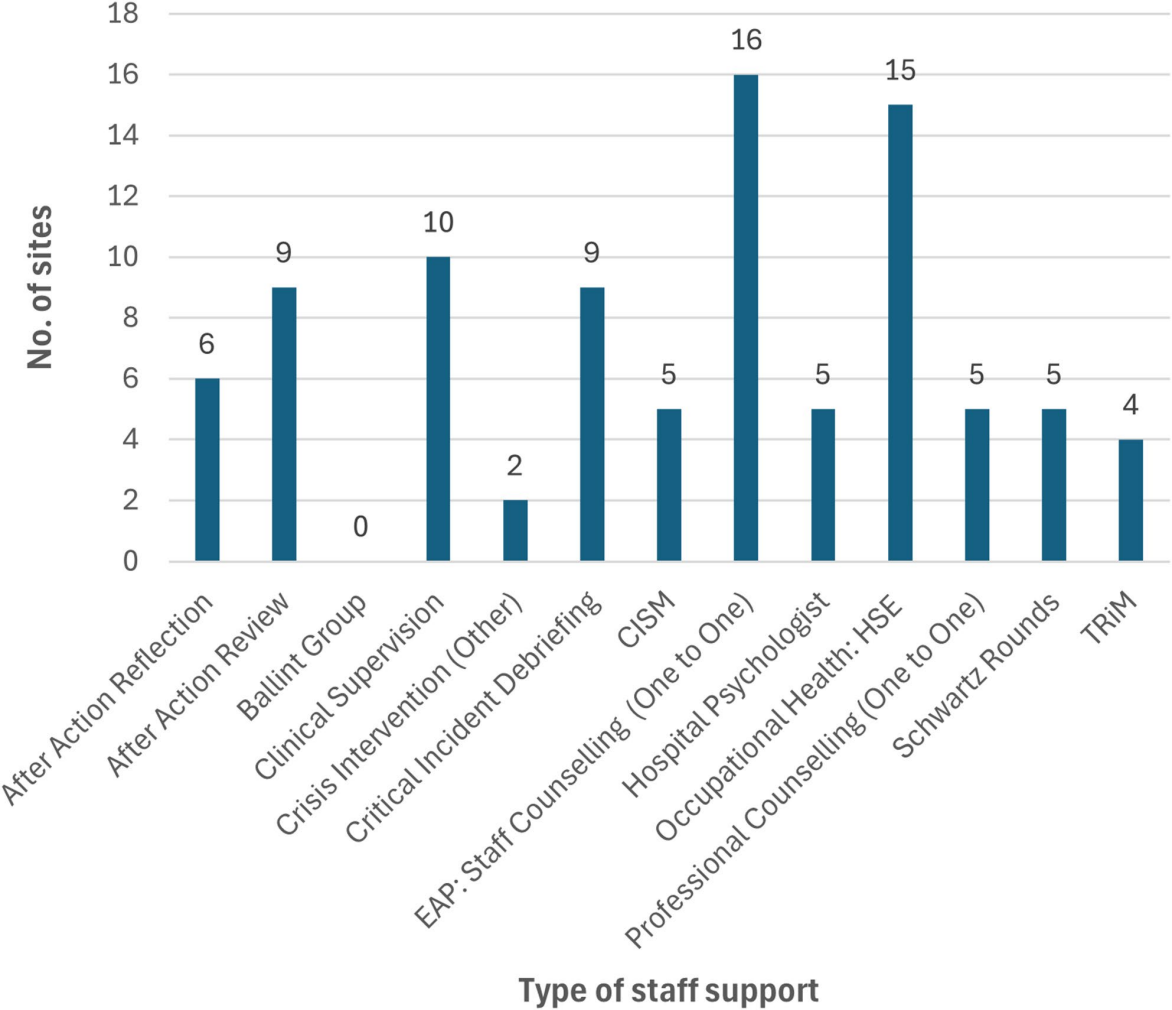
Summary of staff supports across the 18 maternity units

All but one site [Site (S)14] reported the provision of at least one form of psychosocial support offered to staff, with numbers of supports ranging from 2 to 10 (mode = 6); see overview in Table 2; Fig. 1. The most frequently reported supports offered were EAP: Staff Counselling (*n* = 16, 89%); Occupational Health (*n* = 15, 83%); Clinical Supervision (*n* = 10, 56%); After Action Review (*n* = 9, 50%).

**Table 2:** Overview of staff supports across the 18 maternity units

Site ID	Staff supports													
	After Action Reflection	After Action Review	Ballint Group	Clinical Supervision	Crisis Intervention (Other)	Critical Incident Debriefing	CISM	EAP: Staff Counselling (One to One)	Hospital Psychologist	Occupational Health: HSE	Professional Counselling (One to One)	Schwartz Rounds	TRiM	Total no. of supports
S1	No	No	No	No	No	No	No	Yes	No	Yes	No	No	No	2
S2	No	No	No	No	No	No	No	Yes	No	Yes	No	Yes (ND)	No	3
S3	Yes	Yes	No	Yes	No	Yes	No	Yes	No	Yes	No	No	No	6
S4	No^1^	No	No	Yes	No	Yes	Yes	Yes	No	Yes^6^	No	No	No	5
S5	No	Yes	No	No	No	Yes	No	Yes	No	Yes	Yes	Yes	No	6
S6	Yes	No	No	Yes	No	No	No	Yes	No	Yes	No	Yes	No	5
S7	Yes	Yes	No	Yes	Yes (ND)	Yes (ND)	Yes	Yes	Yes	Yes	No	Yes (ND)	No	10
S8	No	Yes	No	No	No	No	No	Yes (ND)	Yes (ND)	No	No	No	Yes	4
S9	No^1^	No	No	Yes	No	Yes	Yes	Yes	No	Yes (ND)	Yes (ND)	No	No	6
S10	No^2^	Yes	No	Yes	No	No	Yes	Yes	Yes	Yes	No	No	Yes	7
S11	No	No	No	Yes	No	Yes	No	Yes	Yes (ND)^5^	Yes	No	No	Yes	6
S12	Yes (ND)	Yes (ND)	No	Yes	Yes	Yes (ND)	Yes (ND)	Yes	No	Yes	No	No	No	8
S13	No^3^	Yes (ND)^4^	No	Yes	No	Yes	No	Yes	Yes	No	No	No	Yes	6
S14	No	No	No	No	No	No	No	No	No	No	No	No	No	0
S15	Yes	Yes	No	No	No	No	No	Yes	No	Yes	No	No	No	4
S16	No	Yes	No	No	No	No	No	No	No	Yes	Yes	No	No	3
S17	No	No	No	No	No	No	No	Yes	No	Yes	Yes	Yes	Unsure	4
S18	Yes	No	No	Yes	No	Yes	No	Yes	No	Yes	Yes	No	No	6
Total (Yes)	**6**	**9**	**0**	**10**	**2**	**9**	**5**	**16**	**5**	**15**	**5**	**5**	**4** ^**7**^	**-**

Notes: ^1^Stated that they provided but detailed EAP/CISM so updated to ‘no’; ^2^Stated that they provided but detailed TRiM so updated to ‘no’; ^3^Stated that they provided but detailed EAP and TRiM so updated to ‘no’; ^4^OBGYN said yes; CMS-BL said no; ^5^Stated that they had a Hospital Psychologist who “Has not made self available to see staff one to one” but is piloting another programme (i.e. TRiM), detailed separately; ^6^MCSF said yes, CMS said no. ^7^This was only available to maternity units within one hospital group when the survey was administered. Abbreviations: ND = no details provided beyond the particular type of support being available

### Characteristics of psychological supports offered

We have divided interventions into four categories for the purpose of presenting the results. For each category, we have summarised details of each of the included interventions below, with supporting details provided in Table 3. Category A includes After Action Reflections, After Action Reviews, and Critical Incident Debriefing as these are generally group-based and, in practice, appear to be conducted to support psychological wellbeing and/or quality improvement or safety, though there was some confusion evident in responses from maternity units in this regard. Category B includes CISM and TRiM as these are specific interventions for psychological well-being following potentially traumatic events and are generally delivered in group format, with potential for individual follow-up. Category C covers more general group-based interventions to enhance psychological wellbeing – Ballint Group and Schwartz Rounds. As none of the 18 sites stated that they provided Ballint Groups, we do not discuss these further. Finally, Category D includes more individual-focused interventions such as Clinical Supervision, EAP, Hospital Psychologist, Occupational Health and Professional Counselling Services.

**Table 3:** Characteristics of psychological supports provided

Support name	Eligible scenarios	Eligible staff	Facilitated or delivered by	Format	Any limits to accessing support
**Category A: After Action Reflections, After Action Reviews and Critical Incident Debriefing**					
After Action Reflection (*N* = 5/6)	• Adverse/traumatic/stressful events or incidents (*n* = 5) [S3, S6, S7, S15, S18], including specific examples of these, e.g. IUD/stillbirth [S15, S18], neonatal death [S6, S7, S18], maternal death [S6, S7, S15], maternal emergency events requiring transfer to ICU [S6], unexpected intrapartum event [S7], or resuscitation [S7, S15]	• All staff; no restrictions (*n* = 5) [S3, S6, S7, S15, S18]	• EAP staff/counsellors (*n* = 2) [S3, S18] • Quality/clinical staff or managers or psychologists (*n* = 2) [S6, S15] • Both (*n* = 1) [S7]	• Varies depending on needs of staff or unstructured (*n* = 5) [S3, S6, S7, S15, S18]	• None (*n* = 5) [S3, S6, S7, S15, S18] • Two sites further noted staff offered one-to-one support (e.g. via EAP) after taking part in After Action Reflection [S6, S18]
After Action Reviews (*N* = 7/9)	• Following an adverse incident, event or outcome (*n* = 7) [S3, S5, S7, S8, S10, S15, S16]. Only two sites provided specific examples, e.g. stillbirth, maternal death [S5, S15]	• Any staff member (*n* = 6) [S3, S5, S8, S10, S15, S16] • “MDT” (*n* = 1) [S7]	• Appropriately trained facilitators (*n* = 6) [S3, S5, S8, S10, S15, S16], with most also specifying either psychologists or therapists, or quality and safety/risk staff [S5, S8, S10, S15, S16] • No response (*n* = 1) [S7]	• Varied information provided; no real sense of format at each site acquired • Most focused on the structure or duration (*n* = 4): “facilitator present, ground rules throughout meeting agreed at the outset” [S3]; “Frequency- as required, duration- usually an hour, but it depends” [S5]; “within 7 days and up to 6 weeks if required” [S10]; “AAR format once off event 1–2 hours” [S16] • Takes place at “Serious Incident management forum” (*n* = 1) [S7] • “Each case is individual” (*n* = 1) [S8] • “Unsure” (*n* = 1) [S15]	• None (*n* = 3) [S3, S5, S8] • Once-off (*n* = 2) [S10, S16], with both noting potential for follow-up support via EAP/line manager • Unsure (*n* = 1) [S15] • No response (*n* = 1) [S7]
Critical Incident Debriefing (*N* = 7/9)	• Primarily following adverse/traumatic events, incidents or outcomes (*n* = 7) [S3, S4, S5, S9, S11, S13, S18]; no specific examples provided	• All staff (*n* = 7) [S3, S4, S5, S9, S11, S13, S18]	• “CSF, CMM3, DOM and consultants” (*n* = 1) [S4] • “Line manager, communication training” (*n* = 1) [S5] • “ADOM lead or QPS lead - varies” (*n* = 1) [S11] • “QPS” (*n* = 1) [S18] • “EAP” (*n* = 1) [S9] • “Trims facilitators” (*n* = 1) [S13] • No response (*n* = 1) [S3]	• Varied information provided • “Meeting between MDT to arrange a debrief for all disciplines involved” / “Monthly meetings” (*n* = 1) [S4] • “informal 1:1 meetings” (*n* = 1) [S5] • “Face to face” (*n* = 1) [S9] • “Varies. Clinical area or classroom. 1–2 hours. Group based. Facilitator leads discussion of the case - what happened, issues, emotions, need for further support” (*n* = 1) [S11] • “As required following SAE. Facilitated by QPS staff member” (*n* = 1) [S18] • Don’t know (*n* = 1) [S13] • No response (*n* = 1) [S3]	• No limit (*n* = 2) [S4, S5] • Once-off debrief (*n* = 2) [S11, S18] • Unknown (*n* = 2) [S9, S13] • No response (*n* = 1) [S3]
**Category B: Critical Incident Stress Management (CISM) and Trauma Risk Management (TRiM)**					
CISM (*N* = 4/5)	• Adverse, traumatic, stressful or unexpected events, incidents and/or outcomes (*n* = 4) [S4, S7, S9, S10]	• All staff (*n* = 4) [S4, S7, S9, S10]	• EAP/CISM-trained staff/therapists (*n* = 3) [S4, S9, S10] • “Clinical Psychologist” (*n* = 1) [S7]	• Structured meetings (*n* = 2) (“structured meeting” [S4]; “structured sessions, approximately 2 weeks after an event with follow on as required” [S7]) • “Face to face” (*n* = 1) [S9] • “Group session with individual session also offered” (*n* = 1) [S10]	• “whenever needed” (*n* = 1) [S4] • “ongoing” (*n* = 1) [S7] • “following serious incident” (*n* = 1) [S10] • Unknown (*n* = 1) [S9]
TRiM (*N* = 4/4)	• Serious and/or potentially traumatic incidents or events [S8, S10, S11] or “following a difficult case or undesired outcome” [S13] (*n* = 4)	• All staff (*n* = 4) [S8, S10, S11, S13]	• Staff specifically trained to deliver TRiM (*n* = 4) [S8, S10, S11, S13], with S13 noting the TRIM team and Clinical Psychologist	• Initial assessment and briefing meeting (for all) [S8, S11] / debrief of events [S13] within 7 days [S10], followed by a structured conversation [S10, S11] for people who may require further support [S13] (*n* = 4)	• Sites did not specify how long TRiM support could be accessed for; one noted 45 min – most likely the duration of initial briefings [S8]. Another noted that it can vary for individuals and groups and can be accessed up to six weeks [S10] • Not aware of any (*n* = 1) [S13] • No response (*n* = 1) [S11]
**Category C: Schwartz Rounds**					
Schwartz Rounds (*N* = 3/5)	• Non-specific answers were provided regarding when or why the support be accessed, with responses reinforcing the open nature of access to the support: “open to everyone” [S5], “self referral, monthly” [S6], “advertised via posters/email” [S17]	• Anyone (*n* = 3) [S5, S6, S17]	• “trained schwartz facilitators” (*n* = 1) [S5] • “Perinatal mental health team” (*n* = 1) [S6] • No response (*n* = 1) [S17]	• Held fortnightly or monthly (*n* = 2): “2 monthly, duration 1 hour” [S5]; “Perinatal mental health team, admin staff, CEO/ Master, monthly” [S6] • No response (*n* = 1) [S17]	• No limit (*n* = 1) [S6] • No response (*n* = 2) [S5, S17]
**Category D: Clinical Supervision, Employee Assistance Programme: Staff Counselling (One-to-One), Hospital Psychologist, Occupational Health, Professional (External) Counselling Services (One-to-One)**					
Clinical Supervision (*N* = 10/10)	• The majority cited specific roles rather than situations in which access was provided: • Bereavement midwives (*n* = 10) [S3, S4, S6, S7, S9, S10, S11, S12, S13, S18] • Perinatal mental health midwives/teams (*n* = 3) [S3, S6, S7] • Chaplains (*n* = 1) [S6] • Medical social workers (*n* = 1) [S6] • SATU staff (*n* = 1) [S9] • Staff psychologist (*n* = 1) [S11]		• Counsellors/psychotherapists (*n* = 7) [S3, S4, S6, S7, S9, S10, S11] • HSE-employed clinical psychologist (*n* = 1) [S13] • “Clinical supervisor with relevant experience” (*n* = 1) [S18] • No response (*n* = 1) [S12]	• Typically reported in terms of duration or frequency, with most stating one hour every month or six weeks (*n* = 7) [S3, S4, S7, S9, S10, S12, S13] • One-to-one sessions (*n* = 3) [S6, S7, S10] • Face-to-face or in-person (*n* = 1) [S9] • Online (*n* = 1) [S11] • Not known (*n* = 1) [S18]	• No limits / ongoing (*n* = 9) [S3, S4, S6, S7, S9, S10, S11, S13, S18] • No response (*n* = 1) [S12]
EAP: Staff Counselling (*N* = 15/16)	• Any professional/personal issue (*n* = 7) [S1, S5, S6, S10, S11, S15, S17] • Following an adverse outcome/incident (*n* = 5) [S2, S3, S7, S13, S18] • Both – adverse event or home issue (*n* = 1) [S4] • Specific adverse events noted most often included stillbirth, with other outcomes reported also: “IUD/stillbirths” [S4]; “SREs adverse outcomes, unexpected outcomes, bereavement, stress” [S7]; “Adverse outcomes in relation to mum & Baby, diagnosis of intrauterine death” [S13]; “SAE, stillbirth/neonatal death” [S18] • No response (*n* = 2) [S9, S12]	• All staff (*n* = 15) [S1, S2, S3, S4, S5, S6, S7, S9, S10, S11, S12, S13, S15, S17, S18], with S1 noting “All HSE employees” and S5 stating “All disciplines”.	• Qualified/trained professionals/psychotherapists (*n* = 3) [S2, S4, S15] • Counsellors/accredited counsellors (*n* = 4) [S5, S10, S11, S13] • External company (*n* = 1) [S6] • Clinical psychologist (*n* = 1) [S7] • EAP (*n* = 4) [S9, S11, S17, S18] • No response (*n* = 3) [S1, S3, S12]	• Counselling [S1, S2] or one-on-one/face-to-face sessions [S6, S7, S9, S11] • Usually up to six sessions [S1 (3–6 sessions); S7 (4–6); S13, S17 (up to 6); S15 (6); S4 (6–8)] provided or “short-term” provision noted [S10]; S15 further highlighted that the six sessions were paid for (i.e. no cost to the staff member) • Sessions were 50–60 min in duration [S13 and S4, respectively], though varied based on individual needs (*n* = 1) [S1] as did frequency or meeting times (*n* = 5) [S1, S4, S5, S11, S12] • No response (*n* = 2) [S3, S18]	• Limits to the number of sessions (*n* = 6) [S1, S5, S9, S10, S11, S17] with some noting the number of sessions (*n* = 4) [4-S5; 6-S9, S10, S17] • No limits (*n* = 5) [S4, S7, S12, S13, S15] • Variation based on individual need (*n* = 3): “pending individual issues” [S6]; “Set number of sessions but can be extended if needed by staff” [S11]; “Relevant staff can continue with individual EAP support after group debrief” [S18] • No response (*n* = 2) [S2, S3]
Hospital Psychologist (*N* = 3/5)	• “SREs, unexpected outcomes, bereavement, stress” (*n* = 1) [S7] • “If staff need follow up following an adverse event” (*n* = 1) [S10] • “for staff as the[y] choose” (*n* = 1) [S13]	• “maternity staff” (*n* = 2) [S10, S13] • “all staff” (*n* = 1) [S7]	• Clinical psychologists (*n* = 3) [S7, S10, S13]	• “only couple/few sessions can be provided due to WTE” (*n* = 1) [S10] • Staff can access “as required and the staff & psychologist discretion” (*n* = 1) [S13] • Unknown (*n* = 1) [S7]	• No limits (*n* = 2) [S7, S13] • “only couple/few sessions can be provided due to WTE” (*n* = 1) [S10]
Occupational Health (*N* = 14/15)	• Most noted that staff could access for any health issue (*n* = 6) [S6, S11, S12, S15, S17, S18], with some further specifying if it impacted on their work performance (*n* = 2) [S11, S18]. • Others noted how: it was available to any/all staff (*n* = 4) [S1, S2, S5, S10] for any area (*n* = 2) [S5, S10] • Sites also reported how access was facilitated as/if needed (*n* = 4) [S2, S3, S4, S16] with S4 specifying “when deemed necessary by CMM2, staff member or GP” • Sites highlighted how it could be accessed (*n* = 3), via self-referral [S6, S17] and/or via line manager [S16] • No response (*n* = 1) [S7]	• All staff (*n* = 13) [S1, S2, S3, S4, S5, S6, S10, S11, S12, S15, S16, S17, S18] • No response (*n* = 1) [S7]	• Dedicated staff/nurses/doctors (*n* = 8) [S4, S5, S6, S7, S11, S16, S17, S18] • Third parties (*n* = 2) (“health and wellbeing unit, Dublin” [S10]); “A 3rd party GP service” [S15]) • No response (*n* = 4) [S1, S2, S3, S12]	• Sites provided varying types of information. • Most generally highlighted the individual(ised) (*n* = 6) [S5, S10, S11, S12, S15, S18] or structured/formal (*n* = 2) [S4, S7] nature of support • Others noted who it was provided by - nurse/doctor (*n* = 2) [S6, S16]. • Unsure (*n* = 1) [S17] • No response (*n* = 3) [S1, S2, S3]	• No limits (*n* = 7) [S6, S7, S10, S11, S12, S15, S18] with S6 and S12 noting “length of employment” [S6] and “as long as needed” [S12] • Individualised nature (*n* = 2) – “on an individual basis” [S5] and “as dictated by occhealth” [S16] • Unsure (*n* = 2) [S4, S17] • No response (*n* = 3) [S1, S2, S3]
Professional Counselling (*N* = 4/5)	Similar responses were reported across these two areas: • Specific roles (*n* = 2) (“Bereavement teams and SATU” [S5]; “Bereavement Cmsp role” [S16]) • Broader access (*n* = 2) (“any professional/personal scenarios” [S17] or “SAE when staff disclose difficulty with mental health issues as a result of their involvement in a SAE” [S18])		• Externally provided by counsellors/psychologist (*n* = 4) [S5, S16, S17, S18]	• “Individual basis” (*n* = 1) [S5] • “Unsure as to format generally every 6–8 weeks” (*n* = 1) [S16] • “counselling one to one” (*n* = 1) [S17] • “As required. 5 sessions approved and paid by hospital.” (*n* = 1) [S18]	• Indefinite or duration of employment (*n* = 2) [S5, S16] • A specific number of sessions (*n* = 2) − 8 [S17]; 5 [S18] (unclear if the latter limit applied to a specific adverse event or time period, or not)

Note: Some sites stated that an intervention was provided, but did not detail any further information; N = No. of sites that detailed information about the support / No. of sites that stated that they provided the support

Abbreviations. CSF: Clinical Skills Facilitator, CMM: Clinical Midwife Manager, Cmsp: Clinical Midwife Specialist, GP: General Practitioner, ICU: Intensive Care Unit, IUD: Intrauterine Death, QPS: Quality and Patient Safety, SAE: Serious Adverse Event, SATU: Sexual Assault Treatment Unit, SRE: Serious Reportable Event, WTE: Whole Time Equivalent

#### Category A: After action reflection, after action review, critical incident debriefing

Within this category, seven sites provided After Action Reflection, nine provided After Action Review and nine provided Critical Incident Debriefing. There appeared to be some confusion regarding interventions within this category. For example, ten sites stated that they provided After Action Reflection; however, on closer examination, two sites detailed information pertaining to EAP/CISM, one site detailed TRiM and one site detailed EAP and TRiM; data for these were recoded under the relevant intervention types. Two sites stated that they provided another form of crisis intervention (Crisis Intervention (other)); however, one site [S7] did not provide any further details. Site 12 noted limited information: that crisis intervention was available to any staff; this support was specific to the maternity service; staff could access via self-referral; access did not require approval by a line-manager; the support is available formally/informally.

After Action Reflection, After Action Reviews and Critical Incident Debriefing were generally available to all staff following adverse, traumatic or stressful events, incidents or outcomes. Some sites mentioned specific scenarios – including stillbirth, neonatal death, maternal death, maternal emergency events requiring transfer to ICU or resuscitation – though mainly for After Action Reflection. The latter may reflect the fact that After Action Reflection was the first support type mentioned in the survey, and respondents did not repeat similar information for each subsequent support. A range of people were involved in the delivery or facilitation of After Action Reflection and Critical Incident Debriefing, including clinical staff, quality and safety staff, and/or counsellors or psychologists. Quality and safety staff, and/or counsellors or psychologists were also involved in delivering or facilitating After Action Reviews with respondents specifically reporting that they were “Appropriately trained facilitators”. Varied information was provided about the structure of each of these three supports, with respondents often noting variation based on the needs of staff in the particular scenario. Respondents noted that there were no limits to accessing After Action Reflections; however, some noted that After Action Reflections and Critical Incident Debriefings were once-off, with others noting no limits. Some respondents further noted that staff could access individual supports (e.g. via the EAP) following After Action Reflections and After Action Reviews.

#### Category B: CISM and TRiM

Five sites provided CISM; however, one [S12] did not provide any further details. Four sites stated that they provided TRiM [S8, S10, S11, S13]. Another site [S17] noted familiarity with TRiM but were unsure if it was available to them or not. Similar to supports within Category A, supports within this category (CISM and TRiM) were made available to all staff following adverse, traumatic or potentially traumatic, stressful, serious or unexpected events, incidents and/or outcomes. These were delivered or facilitated by staff specifically trained or qualified in these areas, including clinical psychologists and therapists. Respondents focused on the structured format of both CISM and TRiM which tended to involve face-to-face contact in a group format shortly after an event, with potential for further individual-level follow-up if further support required. Limited, and often vague, information was provided regarding limits to accessing these supports, with some noting that CISM could be accessed whenever needed [S4], or on an ongoing basis [S7] and one noting that access to TRiM could vary for individuals and groups [S10] and another that they were unaware of any limits [S13].

#### Category C: Schwartz rounds

Five sites stated that they provided Schwartz Rounds [S2, S5, S6, S7, S17]; however, one did not provide any further details [S2] and one stated *that “training has commenced to provide Schwartz rounds in Q2 2024 for all staff”* [S7] and did not provide any further details. S17 stated that they were *“just getting back to Schwartz rounds post covid”* and provided some further details across different questions. Respondents reported that Schwartz rounds could be accessed by any staff at all three sites, with no specific scenarios provided though the varied responses reinforced the open nature of access to the support, e.g. *“open to everyone”* [S5], *“self referral, monthly”* [S6]. Schwartz Rounds were facilitated by *“trained schwartz facilitators” *at Site 5 and the *“Perinatal mental heath team” *at Site 6, every two months (for an hour) and every month, respectively. Only one site responded to the question regarding limits, noting that there was no limit [S6].

#### Category D: Clinical supervision, EAP: staff counselling, hospital psychologist, occupational health, professional counselling

Within this category respondents stated that they provided access to Clinical Supervision (*n* = 10), EAP (*n* = 16), Hospital Psychologist (*n* = 5), Occupational Health (*n* = 15) and Professional (External) Counselling Services (*n* = 5). The majority cited specific roles rather than situations in which access to Clinical Supervision – and to a lesser extent, Professional Counselling – was provided, and this was predominantly bereavement midwives though other roles were also mentioned (e.g. perinatal mental health teams). It should be noted that Clinical Supervision must be undertaken by CMS-BL as part of their role [46], therefore it would be expected that CMS-BL at all sites would have access to clinical supervision; however, only 10/19 reported they did. However, it is also possible that some respondents classified Clinical Provision as ‘Professional Counselling’ and provided details under that instead (e.g. S5, S16). Two of the three sites that provided information noted that staff could access a Hospital Psychologist following adverse events or outcomes. Access to EAP and Occupational Health – while it could be accessed following such events – tended to be broader, available to staff as supports for any personal/professional or health issue, respectively. With the exception of Clinical Supervision, other supports within this category were reported as available to all staff. In general, all of these supports were provided by clinical psychologists, counsellors or psychotherapists; Occupational Health was provided by dedicated staff, doctors and/or nurses. Respondents provided varied levels of information and details about each of the supports in this category, with many noting the one-to-one nature of them, the individualised nature of Occupational Health, and how Clinical Supervision, EAP and Professional Counselling were usually hour-long sessions every 4–6 weeks, with EAP often being limited to a set number of sessions (usually six). There were generally no limits regarding how long staff could access these supports, the exception being EAP and Professional Counselling, for which some respondents noted that there were limits to the number of sessions, e.g. 4–6 in the case of EAP.

### Further details about psychological supports offered

Sites provided limited other details regarding the various supports and therefore information is summarised across all types of supports below.

We asked sites for further information about whether supports were maternity specific or broader, provided formally or informally, accessed via referral or self-referral, if access required line manager approval, how staff were facilitated to attend and how supports were advertised to staff. Details are provided in Additional File 3. In general, there was variation in whether supports were specific to the maternity service or available to staff in the maternity service as part of broader hospital/campus or national supports. TRiM was specific to the maternity service, while CISM was generally available as part of national supports for staff within the health service and EAP and Occupational Health as part of broader hospital/campus supports. All supports were generally formally provided; however, respondents noted formal and informal provisions of After Action Reflection and Critical Incident Debriefing in particular. There was much variation in how supports were accessed across sites, with referral, self-referral and/or automatic referral mentioned. Line manager approval was generally not required to access After Action Review, TRiM, Schwartz Rounds, EAP and Hospital Psychologist, but was required to access Clinical Supervision, with variation reported regarding line manager approval for other forms of support. Staff were generally released to avail of supports during work hours, though, again, variation was reported. Availing of EAP and Occupational Health was more likely in staff’s own time. Respondents reported that staff were made aware of supports in a variety of ways – via line managers in particular, though other ways were reported, including via posters/leaflets, staff meetings or education sessions, email, websites (e.g. HSE), job descriptions (particularly for Clinical Supervision) and induction training (EAP and Occupational Health).

Additional File 4 contains further information regarding materials, costs, reasons for introduction, and any adaptations to supports since they were introduced. In general, respondents stated that they were unable to share materials, with some noting that none existed for some of the supports. A few pointed towards the HSE or FirstLight websites for materials relating to some supports; for example, After Action Reflection (EAP/HSE: S18), After Action Review (HSE: S5; FirstLight: S10), Critical Incident Debriefing (HSE: S5) and EAP (HSE: S10, S11). Regarding costs, most respondents stated that there were no costs associated with any of the supports and/or that there were no costs to staff, and that the HSE or their hospital or hospital group covered the costs. Little insight into why and when particular supports were implemented was garnered. Respondents often stated that it was in response to an adverse event and/or the need to better support staff, or the introduction of a particular policy or framework, or merely noted the year of introduction. Regarding Clinical Supervision, respondents generally noted the year of introduction, which often coincided with when a CMS-BL post was introduced or commenced. Most respondents also stated that the various supports provided had not been adapted since they were introduced or that they did not know if they had been adapted or not.

### Uptake, evaluation and perceived impact of staff supports

A summary of respondents’ views experiences and views of the uptake, evaluation and perceived impact of each of the different types of staff supports is provided in Table 4, along with illustrative quotes. Across each of these areas, respondents provided limited information and therefore we will discuss results across categories as common patterns are observed. As TRiM was only recently introduced at the time of survey completion, respondents were unable to provide much information as it was *“too early to say”*.

Responses varied across sites regarding uptake of the various supports provided. In general, a mix of good and ‘don’t know’ (as data not recorded and/or available) was reported. Varied uptake was further noted for After Action Reviews and Critical Incident Debriefing alongside these responses. Lack of knowledge of uptake of EAP and Occupational Health was more pronounced with many sites reporting that these were confidential services. The majority of sites reported that the various staff supports provided were not evaluated, or that they did not know about evaluation. A very small number of sites mentioned that particular supports were informally evaluated, e.g. through verbal feedback from staff.

Respondents noted general positive impacts of the various staff supports, including on staff wellbeing and morale, or that they were well-received by staff. Some noted more specific impacts. For example, some noted that After Action Reflections, After Action Reviews and CiSM provided a useful opportunity for discussion, reflection and identify areas for improvement. Again, respondents often reported that they did not know or did not provide a response regarding the impact of EAP and Occupational Health, often stating that these were confidential services. Very few negative impacts or observations were reported. Two respondents commented on the lack of awareness of EAP as a staff support or its low uptake [S2, S13], while one noted how staff can be concerned when they have been advised they have been referred to Occupational Health [S15].

**Table 4:** Uptake and evaluation of staff supports

Support name	Uptake	Evaluation	Impact	Illustrative quotes
**Category A: After Action Reflections, After Action Reviews and Critical Incident Debriefing**				
After Action Reflection (*N* = 5/6)	• “midwives, nurses, admin staff, HCA’s, student midwives, Neonatologist, Anesthetics, NCHDs” (*n* = 1) [S6] • “Anecdotally, evidence suggests that uptake is good for both EAP and hospital debrief sessions” (*n* = 1) [S18] • Not recorded (*n* = 1) [S7] • Unknown (*n* = 2) [S3, S15]	• No (*n* = 4) [S3, S6, S7, S15] • No formal evaluation (*n* = 1) [S18]	• Good, non-specific (*n* = 2) [S7, S18] • Useful opportunity to reflect or identify areas for improvement or discuss outcomes (*n* = 3) [S3, S6, S15] • Peer support (*n* = 1) [S6] • Well-received by staff (*n* = 3) [S3, S7, S15]	Evaluation • “Not evaluated formally. But good feedback from staff generally” (*n* = 1) [S18] Impact • “brought team together in supporting each other, removed blame, team identified areas of care for improvement” [S6] • “Good in general but media coverage of adverse outcomes has had a negative effect on staff health & wellbeing” [S18]
After Action Reviews (*N* = 7/9)	• Good uptake (*n* = 3) [S5, S8, S10] • Varies depending on the event/incident, staff involved and/or how soon the After Action Review occurs following the event (*n* = 2) [S15, S16] • No response (*n* = 1) [S7] • Unknown (*n* = 1) [S3]	• Informal: “Verbal feedback” (*n* = 1) [S10] • No (*n* = 5) [S3, S5, S8, S15, S16] • No response (*n* = 1) [S7]	• Direct impacts on staff-opportunity to discuss poor outcomes, feeling listened to, improving morale, enhancing presenteeism, positive impact on staff wellbeing (*n* = 4) [S3, S5, S8, S10, S15] • Potential of such support to enhance patient care (*n* = 1) [S3] • Unsure (*n* = 1) [S16] • No response (*n* = 1) [S7]	Uptake • “approx. 90%” [S5] • “Very well received” [S8] Impact • “Staff feel listened to, voices are heard, staff not enthusiastic re attendance” [S5] • “Support to staff has improved morale, less absenteeism and presenteeism” [S8] • “Unsure maybe of benefit EAP also assists staff” [S16]
Critical Incident Debriefing (*N* = 7/9)	• Varies (*n* = 3) [S4, S5, S11] • Don’t know (*n* = 3) [S9, S13, S18] • No response (*n* = 1) [S3]	• Formal/informal (*n* = 1) [S4] • No (*n* = 3) [S5, S11, S13] – of which one stated that it had just been introduced [S13] • Don’t know (*n* = 2) [S9, S18] • No response (*n* = 1) [S3]	• Feedback generally positive-no specifics provided [(*n* = 3) S4, S11, S18] • Don’t know (*n* = 2) [S9, S13] • No response (*n* = 2) [S3, S5]	Uptake • “not regularly utilised” / “Good uptake” [S4] • “depends on the scenario” [S5] • “Good for some events when well organized and well run” [S11] Impact • “helps the MDT debrief after a case and can highlight learning outcomes” [S4] • “not evaluated but staff feedback has been positive on some occasions” [S11]
**Category B: Critical Incident Stress Management (CISM) and Trauma Risk Management (TRiM)**				
CISM (*N* = 4/5)	• Good (*n* = 2) [S4, S10] • Not known (*n* = 2) [S7, S9]	• No (*n* = 1) [S10] • Unsure (*n* = 3) [S4, S7, S9]	• Good (*n* = 3) [S4, S7, S10], with one emphasising the benefit of space to process an event/discuss concerns [S4] • Not known (*n* = 1) [S9]	Uptake • “[CISM is] commonly arranged for staff after traumatic events” [S4] Impact • “very helpful to staff for them to process the event; very beneficial provides a safe confidential space for staff to discuss their concerns” [S4]
TRiM (*N* = 4/4)	• Only newly available to staff and, as such, it was *“too early to say”* [S11] (*n* = 4) [S8, S10, S11, S13] • S10 also noted that *“up to 15*,* all staff [types]”* had availed of TRIM.	• Formal (*n* = 1) [S8] • Pilot at present - evaluation after 3 months (*n* = 1) [S10] • No (*n* = 1) [S11] • Not known (*n* = 1) [S13]	• Staff find helpful, value being cared for (*n* = 2) [S8, S13] • Better approach to supporting staff (*n* = 1) [S10] • Too early to say (*n* = 1) [S11]	Impact • “Successful and staff found same helpful.” [S8] • “staff being valued and cared for” [S10] • “[TRiM is a] Better and a more formal approach to supporting staff after a difficult incident” [S13]
**Category C: Schwartz Rounds**				
Schwartz Rounds (*N* = 3/5)	• Good, across a range of staff types and students (*n* = 1) [S6] • Unsure (*n* = 1) [S17] • No response (*n* = 1) [S5]	• Yes; formally (*n* = 1) [S6] • Unsure (*n* = 1) [S17] • No response (*n* = 1) [S5]	• “staff wellbeing, morale” (*n* = 1) [S6] • Unsure (*n* = 1) [S17] • No response (*n* = 1) [S5]	Uptake • “20 to 30 staff attend a session, mainly nurses, midwives, NCHD’s, HCA, admin staff, management staff, catering and household staff, students, neonatologist” [S6]
**Category D: Clinical Supervision, Employee Assistance Programme: Staff Counselling (One-to-One), Hospital Psychologist, Occupational Health, Professional (External) Counselling Services (One-to-One)**				
Clinical Supervision (*N* = 10/10)	• Limited access to Clinical Supervision, i.e. only available to specific roles (*n* = 2) [S3, S13] • Accessed by all those to whom it is available (*n* = 4) [S4, S6, S10, S11] • Don’t know (*n* = 2) [S9, S18] • No response (*n* = 2) [S7, S12]	• No (*n* = 7) [S3, S4, S6, S10, S11, S13, S18] • Don’t know (*n* = 1) [S9] • No response (*n* = 2) [S7, S12]	• Positive impacts, particularly on staff-wellbeing and clinical practice (*n* = 8) [S3, S4, S6, S9, S10, S11, S13, S18] • No response (*n* = 2) [S7, S12]	Uptake • “used by the limited staff to whom it is deemed to apply” [S11] Impact • “positive impact for staff and patients” [S3] • “Successful when implemented right” / “Every aspect of Bereavement Midwife role” [S4] • “staff wellbeing, guidance, education, reflection” [S6]
EAP: Staff Counselling (*N* = 15/16)	• Generally good (*n* = 1) [S18] • Low uptake implied (*n* = 1) [S2] • Don’t know / confidential service (*n* = 10) [S1, S4, S5, S6, S7, S9, S11, S13, S15, S17] • No response (*n* = 3) [S3, S10, S12]	• Informally evaluated (*n* = 1) [S4] • Not evaluated (some specifying locally/at hospital level) (*n* = 4) [S6, S9, S17, S18] • Don’t know (*n* = 4) [S7, S11, S13, S15] • No response (*n* = 6) [S1, S2, S3, S5, S10, S12]	• Positive impacts on staff health/wellbeing (*n* = 2) [S4, S18] • Positive feedback from staff (*n* = 2) [S6, S7] • “a good support service” (*n* = 1) [S10] • Implied impact not as good as it could be-low uptake (*n* = 1) [S13] • Don’t know (*n* = 4) [S9, S11, S15, S17], with three further noting that attendance was confidential • No response (*n* = 5) [S1, S2, S3, S5, S12]	Uptake • “A lot of staff are unaware it exists” [S2] • “[I] encourage and recommend EAP” [S1] • “unknown as staff dont need to inform anyone of their engagement with EAP” [S17] Impact • “I think the uptake is low for EAP” [S13] • “Generally, good impact on staff health and wellbeing” [S18]
Hospital Psychologist (*N* = 3/5)	• “good uptake to bitesize workshops” (*n* = 1) [S10] • Don’t know (*n* = 2) [S7, S13]	• “no - new service since May 2022” (*n* = 1) [S10] • Don’t know (*n* = 2) [S7, S13]	• Good feedback (*n* = 1) [S10] • “benefit as gives staff a confidential space” (*n* = 1) [S13] • Don’t know (*n* = 1) [S7]	Impact • *“*new service since May 2022 - good verbal feedback” (*n* = 1) [S10]
Occupational Health (*N* = 14/15)	• Very positive (*n* = 1) [S6] • Generally good (*n* = 1) [S18] • “Only refer if necessary. long term illness” (*n* = 1) [S15] • Unsure or don’t know as services is confidential (*n* = 4) [S7, S10, S11, S17] • No response (*n* = 7) [S1, S2, S3, S4, S5, S12, S16]	• Yes (*n* = 1) [S10] (“continuously evaluated”) • No (*n* = 4) [S6, S15, S16, S18] • Don’t know (*n* = 3) [S7, S11, S17]. • No response (*n* = 6) [S1, S2, S3, S4, S5, S12]	• Positive impacts on staff and/or staff wellbeing/morale (*n* = 5) [S4, S6, S7, S10, S18] • Staff concerned (*n* = 1) [S15] • No evaluation (*n* = 1) [S16] • Don’t know (*n* = 2) [S11, S17] • No response (*n* = 5) [S1, S2, S3, S5, S12]	Uptake • “Generally good uptake when required and referral made” [S18] Impact • “Staff can be concerned when they have been advised they have been referred” [S15] • “Generally, good feedback and staff agree with recommendations” [S18]
Professional Counselling (*N* = 4/5)	• Don’t know (*n* = 2) [S17, S18] • “Depends on service” (*n* = 1) [S5] • “1 staff member only” (*n* = 1) [S16]	• No (*n* = 3) [S5, S16, S17] • No response (*n* = 1) [S18]	• Impact *“Specific to one role”* (*n* = 1) [S16] • Unsure (*n* = 1) [S17] • No response (*n* = 2) [S5, S18]	None (no additional illustrative quotes)

Abbreviations. HCA: Health Care Assistant, MDT: Multi-disciplinary Team, NCHD: Non Consultant Hospital Doctor

## Conclusions

Maternity staff commonly experience adverse events, which can have profound impacts [9, 12], and require the provision of appropriate psychosocial supports [25, 27]. In this study, we mapped such supports within 18 of the 19 maternity units (sites) in the Republic of Ireland. All but one reported the provision of at least one form of psychosocial support for staff, with numbers of supports ranging from 2 to 10. The most frequently reported supports offered were generic, i.e. not specific or limited to adverse events, namely EAP (*n* = 16) and Occupational Health (*n* = 15). Clinical Supervision was available at 10 sites, but was specific to particular roles, primarily CMS-BL. As noted, Clinical Supervision is a requirement of this role, and is an important strategy in enhancing the wellbeing and resilience of midwives [47].

There appeared to be confusion between some types of supports, namely those within categories A and B. For example, sites may have provided information about CISM and/or TRiM but also provided similar information under Critical Incident Debriefing. This is perhaps reflective of the broader literature regarding clinical debriefing which notes that while it is widely used in practice, it lacks standardisation and agreed best process [48]. While there are differences between different forms of psychological intervention types – such as clinical debriefing, After Action Reviews and Critical Incident Stress Debriefing, there is some overlap [49]. Terms such as debrief, After Action Review, after-event review, guided team self-correction, and reflexivity, are often used to describe the same approach [50]. Debriefing methodologies or interventions, including ‘Critical Incident Stress Debriefing’ and ‘Critical Incident Stress Management’ (such as CISM and TRiM), instead aim to provide psychological support [49]. They can, however, have varying goals, including emotional or psychological support and system or procedural learning or improvements [51]. Furthermore, a recent Irish study found that staff may informally facilitate After Action Review but might not characterise it as such for fear of lack of engagement [52], which may also account for some of the confusion we observed. All of this may have implications for staff who could benefit from such supports. They may not understand what the different supports entail and/or have preconceived ideas, which may be misaligned with the intended aims of these supports, resulting in a lack of uptake or reported benefit. It must be acknowledged that a range of interventions can occur following an adverse event to address different needs [49]. Furthermore, limited numbers of staff provide psychosocial supports; this should be borne in mind when deciding on the appropriate mix of staff supports within a given setting.

Only 9 of the 18 sites provided After Action Review despite this being a specified support under the National Incident Management Framework [25]. While healthcare staff across the Irish health services have been trained as After Action Review facilitators [53], some barriers to their implementation of this support have been documented [52]. After Action Reviews can take various forms. They are not generally intended as a psychological support for staff, instead providing the opportunity for staff to reflect on an event in a manner that focuses on learning and improvement [30, 49, 54]. That said, After Action Reviews within the HSE Incident Management Framework are thought to enhance safety culture and staff healing from the impact of errors [25].

While sites generally stated what supports were provided, they provided little detail on these, particularly regarding uptake, evaluation and impact. Thus, while these supports may be available, staff may not be aware of them or what they entail, or have the opportunity or motivation, to access them, despite the various forms of advertising reported across sites. In their study of maternity care professionals in Ireland, McNamara and colleagues [8] observed that, while 80% agreed that debriefing would be beneficial after experiencing an intrapartum death, only 11% were offered a chance to formally debrief with colleagues. Similarly, a survey of midwives in Spain found that 81% had no form of specialised support in their workplace to support professionals who assist women with perinatal loss [55]. Lack of awareness of the availability and/or access to After Action Reviews has been noted more generally within the health services, with lack of visibility an identified implementation barrier [52]. Based on the results of our study, there is room for standardisation and improvements in supports provided, including the advertising and awareness-raising of these.

### Strengths and limitations

Our study provides an overview of psychosocial supports for maternity care staff following adverse events in the Republic of Ireland. A strength of our study is that we received responses regarding staff supports from 18 of the 19 maternity units in the jurisdiction. The survey was administered alongside a survey of perinatal bereavement education and training, with questions relating to staff supports in the second part of the survey, which may have affected the quantity of information provided. It may also be reflective of a lack of in-depth knowledge of the nature or uptake, evaluation and perceived impact of staff supports by those who completed the survey. Data was provided by the person and/or teams responsible for staff education and training and supports; it may be limited to the knowledge or perceptions they hold within their respective roles. Thus, it may not reflect the breadth and/or depth of psychosocial supports provided, or details thereof, despite the research team advising that responses should reflect overall provision. The majority of respondents were CMS-BL. We may have received different information regarding staff supports from staff within human resources / staff wellbeing, quality and patient safety, or other roles. That said, units were asked to nominate those who were best placed to provide the required information on behalf of their unit and, as noted earlier, we are aware that some responses were prepared by a team of people and submitted by one person. We did not provide definitions of After Action Reflection and Critical Incident Debriefing (though noted this was different to CISM and TRiM) within the survey, which may have contributed to some confusion between the different types of supports within these categories. Furthermore, we did not independently verify the accuracy of information provided; neither did we seek broader staff views of such supports.

In this study, we have generated an initial mapping of psychosocial supports for staff within maternity units in the Republic of Ireland following adverse events. Efforts to enhance the uptake and evaluation of such supports in practice are needed to maximise the impacts and outcomes for staff who could benefit from them, given the frequency with which they encounter adverse or traumatic events and the deficits in supports reported. In addition, further research is required to better understand the nature, impacts and implementation of such supports to inform future development and potential scale-up of supports nationally.

## Supplementary Information

Below is the link to the electronic supplementary material.

Supplementary Material 1

Supplementary Material 2

Supplementary Material 3

Supplementary Material 4

## Data Availability

The data that support the findings of this study are not openly available due to reasons of sensitivity and are available from the corresponding author upon reasonable request.

## Abbreviations

CISM: Critical Incident Stress Management
CMS: Clinical Midwife Specialist
EAP: Employee Assistance Programme
HSE: Health Service Executive
TRiM: Trauma Risk Management
