# Supplementary material for: Psychosocial supports for staff in maternity hospitals and units following adverse events: a mapping study in the Republic of Ireland
Source: BMC Health Serv Res. 2026 Mar 30;26:672. doi: 10.1186/s12913-026-14465-7 (PMC13159316; doi:10.1186/s12913-026-14465-7)
Supplement: Supplementary file 1 — Supplementary Material 1 [file 12913_2026_14465_MOESM1_ESM.docx]

Additional File 1. Survey

Start of Block: Block 1_Consent

Pregnancy loss and perinatal death:

Education, training and support for staff working in maternity hospitals

You are being asked to participate in a study about education and training opportunities on bereavement care, and supports following adverse perinatal and/or maternal events for staff within maternity services. To decide whether you want to take part, you should have enough information to make an informed decision. Please read this information carefully and discuss it with others if you wish.

**What is the purpose of this study?** The aim of this study is to establish, for the 19 maternity units/hospitals in the Republic of Ireland, the extent and nature of the provision of: education and training opportunities for staff on bereavement care for pregnancy loss, as well as supports for staff following adverse events, focusing on pregnancy loss. Findings from this study will be used to inform efforts to enhance the implementation of the National Standards for Bereavement Care for Pregnancy Loss and Perinatal Death. The study will provide updated information on the types of education/training and supports that are (or have been) available in the 19 maternity units/hospitals. This will inform discussions around the development or adaptation and potential scale-up of programmes/interventions nationally.

**Who is conducting this study?** This study is being conducted by a team of researchers within the Pregnancy Loss Research Group at the INFANT Centre, University College Cork (UCC) and Cork University Maternity Hospital (CUMH). The study is led by Dr Keelin O’ Donoghue, Professor at UCC and Consultant Obstetrician at CUMH. It is supported by the Irish Research Council and the National Women and Infants Health Programme.

**What does the study involve?** We are asking you to complete an online survey about the provision of education and training around bereavement care following pregnancy loss and perinatal death, and supports for staff following adverse events, within your maternity unit/hospital; you may also opt to complete the survey with a member of the research team via Microsoft Teams, over the telephone, or in-person. The survey comprises mainly open-ended questions and should take about 45-60 minutes to complete; this may vary depending on the level of detail you provide. On completion of the online survey, we may ask you to clarify, or provide further information about your responses, via interview or email; we will seek your permission for this.

**Can I change my mind?** You can withdraw from the study, without giving a reason, at any time – up to the point of publication – by notifying a member of the research team of your decision.

**What are the possible risks and benefits of taking part?** Taking part will give you the opportunity to provide information about the education/training and supports you provide for staff in relation to pregnancy loss, perinatal death and adverse events. You may not benefit personally; however, the results will be used to inform the development of staff education and training programmes and supports in the Republic of Ireland.

We don’t envisage any negative consequences for you. Any write-up of results of the survey will not identify you, or your unit/hospital, and will be confidential to the research team. The aim of the study is to establish what is currently provided nationally, not to evaluate practices within individual hospitals or units and/or by individual participants.

**What will happen to the information I provide?** UCC is the Data Controller for this project and owns the information collected as part of the study. If you take part, information about you (also called “personal data”) will be accessed, collected and stored for the purposes of this study. This personal data includes information that directly identifies you (such as your name, email address, telephone number and the name of your unit/hospital), and information on your role. It will be treated with the highest standards of security and confidentiality, in accordance with Irish and European Data Protection legislation.

To ensure confidentiality, the data generated during the study is coded with a number that will identify you in the study. Any information that leaves the study site will be labelled with your code instead of your name. A list or ‘key’ linking your study number to your name will also be kept securely by the research team, in a separate password-protected electronic file on secure servers at UCC. Please be assured that your personal data will be accessible to researchers involved in this study only, it will be password-protected and stored electronically on secure servers at UCC. Your data will be retained for ten years after the completion of the project.

Any personal data you provide to us during the course of this study will be processed fairly and lawfully. The research team will use your personal data within the purposes outlined above. If the research team wishes to use your data for a purpose other the purpose specified, they must contact you again to give you more information and ask your permission to use your data for the new purpose. The General Data Protection Regulation (GDPR) allows us to process your data because the research is of substantial public interest (Articles 6(1) (e) and 9(2) (j) of the GDPR). If you require further information on the legal basis for processing your personal data, please contact UCC’s Data Protection Officer – details below.

**Will this be confidential?** Your participation in this study and any personal information provided will be managed with the strictest of confidence. You or your maternity unit/hospital will not be named or identifiable in any materials arising/published from this study.

**What will happen to the results of the study?** Pseudo-anonymised results from the study will be published in international peer-reviewed journals, shared at research meetings and conferences, and may be used for teaching purposes. A summary report of the findings will be shared with the National Women and Infants Health Programme and sent to the 19 maternity units/hospitals across the country and professional bodies/colleges. We will also share this report with you, if you wish to receive a copy.

**Is this study approved by an ethics committee?** Yes, the Clinical Research Ethics Committee of the Cork Teaching Hospitals (CREC) have granted ethical approval for this study. If you have any concerns about this study, you may contact CREC on 021-4901901 or crec@ucc.ie.

**What are my rights?** You have various rights under data protection law, subject to certain exemptions, in connection with our processing of your personal data, including the right:

- To find out if we use your personal data, access your personal data and receive copies of your personal data
- To have inaccurate/incomplete information corrected and updated
- In certain circumstances, to have your details deleted from systems that we use to process your personal data or have the use of your personal data restricted in certain ways
- To object to certain processing of your data by UCC
- To exercise your right to data portability where applicable (i.e. obtain a copy of your personal data in a commonly used electronic form)
- To withdraw your consent to the processing of your data at any time without giving a reason by notifying your decision to the investigator. This will not affect the lawfulness of processing data about you based on your consent before the withdrawal. If you withdraw your consent for data processing, your participation in the study stops and no further data will be collected from you. A researcher will present you the options you have concerning your personal data
- Along with study withdrawal, you have the right to request the deletion of data about you if your data are no longer necessary for the purposes of processing or there is no other legal ground for their further processing.

If you wish to exercise any of these rights, please address your request to the research team or the Data Protection Officer, University College Cork (details below).

**Do I have to sign anything?** If you agree to take part in the study, you will be asked to indicate your consent in the next section (consent form). This is to show that you have understood what is involved and that you have read the Information Sheet. You still have the right to withdraw from the study at any time. If you decide to participate but then change your mind before finishing the survey, simply close your web browser.

**Who can I contact about this study?** **If you have any questions about this study** please contact Marita Hennessy (maritahennessy@ucc.ie) or Professor Keelin O’Donoghue (k.odonoghue@ucc.ie).

**If you have any complaints in connection with our processing of your personal data**, you can contact UCC’s Data Protection Officer (DPO): DPO, Information Compliance Section, Office of Corporate & Legal Affairs, University College Cork, Western Road, Cork E: gdpr@ucc.ie

**You also have the right to lodge a complaint with the Data Protection Commission if you are unhappy with our processing of your personal data.** Details of how to lodge a complaint can be found on the Data Protection Commission’s website (www.dataprotection.ie), or by telephoning 1890 252 231.

| Page Break |  |
| --- | --- |

**CONSENT**
 
Please read each of the following statements carefully and indicate your agreement to participate in the “Pregnancy loss and perinatal death: Education, training and support for staff working in maternity hospitals" study, below.
 
I confirm that:

- I have read and understood the information sheet for the “Pregnancy loss and perinatal death: Education, training and support for staff working in maternity hospitals” study, received a copy, and had the opportunity to ask questions
- I agree to participate in an online survey and follow-up interview (where necessary) to provide information about education and training opportunities on bereavement care, and staff supports
- I give permission for my interview to be audio recorded, and transcribed verbatim by a transcriptionist and/or automatically via Microsoft Teams
- I understand that participation in the study is voluntary and that I am free to withdraw without conseuence, at any time, without giving a reason
- I agree to the publication of anonymised data from my survey/interview in reports, presentations and other publications
- I understand that I can request a copy of my personal information at any time by contacting the research team
- I understand that confidentiality of records concerning my involvement in the study will be maintained in an appropriate manner
- I understand that, when required by law, the records of this research may be reviewed by government agencies and sponsors of the research
- I understand that I can contact the research team at any time about this evaluation: Marita Hennessy, PhD (maritahennessy@ucc.ie); Professor Keelin O'Donoghue (k.odonoghue@ucc.ie)
- I understand that the study has been approved by the Clinical Research Ethics Committee of the Cork Teaching Hospitals (CREC) and if I have further queries concerning my rights in connection with the research, I can contact the CREC at Lancaster Hall, 6 Little Hanover Street, Cork by email at crec@ucc.ie
- I agree to be contacted about this research in the future.

**Please tick here if you wish to participate in this study**. If you do not wish to participate, please close your web browser now.

- Yes, I would like to participate

End of Block: Block 1_Consent

Start of Block: Block 2_Instructions

**Instructions for completing the survey**

**About this survey**

The aim of this survey is to establish, for the 19 maternity units/hospitals in the Republic of Ireland, the extent and nature of the provision of: education and training opportunities for staff on bereavement care for pregnancy loss, as well as supports for staff following adverse events, focusing on pregnancy loss. This will inform discussions around the development or adaptation and potential scale-up of programmes/interventions nationally.

**How to complete the survey** Please tell us as much as you can for each question – write what immediately comes into your head and/or take time to reflect on what you want to say. There are no right or wrong answers. If you need time to think about the questions/your responses, you can leave the survey and come back to it again; you don’t have to complete it in one sitting.

You may need to consult with other members of staff to answer some of the questions. If you leave the survey, your responses will be automatically saved, and the survey link will allow you to return to where you left off (on the same device). You can return to edit your previously completed responses at any stage before submitting the completed version.

**If you have any questions** about the survey, at any stage, please contact Marita Hennessy, Postdoctoral Researcher within the Pregnancy Loss Research Group (maritahennessy@ucc.ie).

End of Block: Block 2_Instructions

Start of Block: Block 3_Section A: About you/your unit

**SECTION A: About you and where you work**
In this section, we want to find out about you and your background. If several people from the hospital contribute to this survey response, this section should be completed by the person filling out the survey on behalf of the maternity unit/hospital.

**Maternity unit/hospital name**. Please select from the drop down menu

▼ Cavan Monaghan Hospital (4) ... Wexford General Hospital (25)

**Details of person completing the survey on behalf of the maternity unit/hospital**.

Note: We will keep your name and contact details confidential; we will only use them to contact you if any further information is needed.

Name _______________________________________________________________________

Job title _____________________________________________________________________

Role in relation to staff education and training and supports _____________________________

Email address ________________________________________________________________

Telephone number ____________________________________________________________

**NOTE:** If you would prefer to complete the survey by interview with a member of the research team – virtually (via Microsoft Teams), over the telephone, or in-person – please contact Marita Hennessy, Postdoctoral Researcher within the Pregnancy Loss Research Group (maritahennessy@ucc.ie).

Some of the responses may be time consuming to complete; you may find that completing the survey with a member of the Research Team may be more time efficient.

End of Block: Block 3_Section A: About you/your unit

Start of Block: Block 4_Section B_Staff training

**SECTION B: Staff education and training on bereavement care surrounding pregnancy loss and perinatal death**
In this section, we want to find out about education and training provided to staff within your maternity unit/hospital – which specifically relate to bereavement care surrounding pregnancy loss and perinatal death.

**Please tell us about the types of education and training on bereavement care you provide or that staff can access within your maternity hospital/unit. We are particularly interested in learning about programmes you provide on-site/in collaboration with others (e.g. within your hospital group). We are focusing on hospitals/hospital groups in this survey, because we need your help to get this information.**

Note: For this survey, we are excluding stand-alone events/meetings organised by national training bodies, professional colleges and organisations - such as the Royal College of Physicians in Ireland (RCPI), National Women and Infants Health Programme (NWIHP), National Perinatal Epidemiology Centre (NPEC), and Irish Hospice Foundation (e.g. national Hospice Friendly Hospitals network meetings) - and universities.

We have provided space for you to enter details of up to 8 programmes. If you provide more than this, please contact Marita Hennessy PhD (maritahennessy@ucc.ie) for further guidance. Please tell us as much as you can about each programme.

| Page Break |  |
| --- | --- |

**Programme 1** [This format was duplicated to enable participants to detail up to 8 programmes (Programme 2, Programme 3, etc.; not replicated here, for brevity]

**Do you wish to provide details about a staff education/training programme here?**

We are asking you this question so that we can direct you through this online survey as efficiently as possible. If you do not wish to provide details of a staff training/education programme here then you will be directed to the next section of the survey.

- Yes
- No

Name of the education/training programme

________________________________________________________________

Aim of the programme

________________________________________________________________

Who are the target audience for this programme? Please detail types of staff eligible – medical (doctors/midwives/nurses), allied health, other. Include trainees where applicable also.

________________________________________________________________

Are there any prerequisites to attending this education/training programme? E.g. certain qualifications have to be held. If so, please detail

________________________________________________________________

Is this staff education/training programme provided...

|  | Yes | No |
| --- | --- | --- |
| Locally, within your unit/hospital |  |  |
| Regionally, within your hospital group/other geographical arrangement. Please provide details ____________ |  |  |
| Nationally |  |  |
| Other, please specify |  |  |

Who provides/offers (validates/certifies) this education/training? Please select all that apply.

- Centre of Nursing and Midwifery Education
- Hospital staff
- Irish Hospice Foundation
- Nursing and Midwifery Planning and Development Unit
- Royal College of Physicians of Ireland
- Another charity, please provide their name __________________________________________________
- Other, please specify __________________________________________________

Do you make it mandatory for staff to attend this education/training programme?

- Yes
- No

Who facilitates/delivers this education/training: Please specify who; own staff/external facilitator(s); training/qualifications to deliver; any supports for those who deliver training

________________________________________________________________

How is the education/training delivered? Please select one only

- In-person / Face-to-face
- At a distance / virtual / online
- Hybrid

Where is the education/training delivered? Please select one only

- On-site
- Off-site, please specify where __________________________________________________
- On-site and off-site, please specify where off-site __________________________________________________
- Other, please specify (e.g. online-can participate from any location) __________________________________________________

Please provide a brief overview of the topics covered in this education/training programme

________________________________________________________________

Please provide an overview of the teaching & learning strategies used (e.g. didactic / interactive, e.g. role play, simulation, practical work-quizzes)

________________________________________________________________

What is the duration of the education/training programme? Please specify no. of hours/days

________________________________________________________________

Is it delivered...? [Please select one option only]

- As a single session
- Over multiple sessions, please insert no. __________________________________________________
- Other, please specify

__________________________________________________

Please specify the timing/frequency of delivery of this education/training programme – when is it delivered, e.g. during induction of new staff / annually / every X months / every X years

________________________________________________________________

Is it once-off education/training for staff, or recurring (e.g. with refresher sessions)

- Once-off
- Recurring, please provide details __________________________________________________

What costs, if any, are associated with the provision of this education/training programme? (e.g. financial, in-kind)

________________________________________________________________

Who covers the costs of providing this education/training programme (e.g. participants themselves and/or hospital/HSE-financed)? Please provide as much detail as you can

________________________________________________________________

Are staff...?

|  | Yes | No |
| --- | --- | --- |
| Paid to attend this education/training programme |  |  |
| Released on a working day to attend |  |  |
| Attend on a day off - paid |  |  |
| Attend on a day off - given time off in lieu |  |  |
| Other, please specify ____________ |  |  |

Is this course/programme accredited?

- Yes
- No

Who is the course/programme accredited by?

|  | Yes | No |
| --- | --- | --- |
| Centre of Nursing and Midwifery Education / Centre of Midwifery Education (CNME / CME) |  |  |
| Continuous medical education (CME) locally |  |  |
| Nursing and Midwifery Board of Ireland (NMBI) |  |  |
| Royal College of Physicians of Ireland (RCPI) |  |  |
| Other, please specify: ___________________ |  |  |

How is the programme advertised/details shared with staff?

________________________________________________________________

Is there a minimum/maximum number of participants needed the run this education/training programme? Please provide as much information as you can

________________________________________________________________

Do you record how many participants take part in this education/training programme, as standard?

- Yes, please specify how/where __________________________________________________
- No

Thinking about the reach of this education/training programme, i.e. who takes part versus who you would like to take part in it ... How many people usually attend, on average, compared to how many could attend/benefit from the programme? In practice, who usually attends this education/training? Are there any particular groups or types of staff that are difficult to reach with this education/training or who do not usually attend (for whatever reason(s))? Please provide as much information as you can

________________________________________________________________

Are any materials used in the delivery of the programme, e.g. workbooks, guidelines, videos, handbooks. If so, please provide details:

________________________________________________________________

Would you be able to share any education/training materials with us? If so, please note details if available online / email copies to maritahennessy@ucc.ie

- Yes
- No

If applicable, where was this education/training programme sourced from/how was it developed (and why this particular programme/approach)?

________________________________________________________________

How frequently is the content/format of this education training programme reviewed/updated?

________________________________________________________________

Have any changes have been made to this education/training programme since it was first offered? If yes, please provide details (what and why):

- Yes, please specify any changes __________________________________________________
- No
- Don't know

If there is anything else you would like to tell us about this staff education/training programme, please note it here

________________________________________________________________

End of Block: Block 4_Section B_Staff training

[Blocks for Programmes 2 to 8 presented, as required]

Start of Block: Block 4_Section B_Final Qs

**Thinking more about staff education and training on bereavement care, are there any education and training programmes that you delivered in the past, but do not deliver any more?**Can you tell us more about these – what programmes, why you stopped delivering them, any plans to re-introduce them?

________________________________________________________________

| Page Break |  |
| --- | --- |

**Similarly, are there any staff education and training programmes that you would like to offer/deliver but currently are not?**Again, can you tell us as much as you can about these – what programme(s), why would you like to offer it, what is stopping you from offering it, what would enable you to offer it?

________________________________________________________________

| Page Break |  |
| --- | --- |

**In your opinion, what works – or does not work – in terms of the provision of staff education and training on bereavement care?**When answering, think about those delivering and receiving such programmes, what is needed to implement such programmes and also what is needed to sustain (keep delivering) such programmes, and what supports or hinders these, within your hospital/unit.

________________________________________________________________

| Page Break |  |
| --- | --- |

**Is staff education and training on bereavement care evaluated in your unit/hospital?**Can you tell us more about this – for what programmes, by who, formally or informally, what type of data is gathered and when (e.g. attendance numbers, feedback surveys), what is done with feedback (e.g. is a report generated and shared with anyone), what kind of feedback is usually received on programmes, etc. Would you be able to share any reports with us? If so, please note details if available online / email copies to maritahennessy@ucc.ie. In your opinion, what has the impact of this training been – on patient care, level of complaints, staff morale?

________________________________________________________________

| Page Break |  |
| --- | --- |

**Are people with lived experience (e.g. of pregnancy loss) involved in the design and/or delivery of any staff education and training on bereavement care?**Can you tell us more about this, e.g. how they are involved and why, what the impact of their involvement is (if any), what supports their involvement. If not involved, are there any particular reasons for this?

________________________________________________________________

End of Block: Block 4_Section B_Final Qs

Start of Block: Block 5_Section C: Staff supports

**SECTION C: Staff supports surrounding adverse events**
**In this section we want to find out about supports/support services that are provided to staff following adverse events within your maternity unit/hospital – these can relate to pregnancy loss/perinatal death, but also more broadly in relation to adverse perinatal/maternal-related events. We are interested in supports for individuals, teams, or all hospital staff, delivered in all formats.**
 
In the following sections, we will ask you if you provide specific named supports/support services and, if so, we will then ask you questions about this support. If you do not provide a particular support, on selecting 'no', you will be directed to the next relevant section. 
 
There may be other supports that you provide that are not listed. If this is the case, we have left space for you to provide details of up to three supports/services. If you wish to detail any further supports, please contact Marita Hennessy (maritahennessy@ucc.ie) for further guidance.

**Are any of the following staff supports/support services available to staff in your maternity unit/hospital?** Please select yes or no to each type of support listed below and you will be directed through the survey accordingly.

**After action reflection**

- Yes
- No

Display: If After action reflection = Yes

**After action reflection** In what clinical, professional or work scenarios does access to this support apply or become available? Please provide as much detail as you can (e.g. detail specific types of outcomes, incidents or events)

________________________________________________________________

Who can avail of this support – any restrictions by speciality, status (staff – healthcare or allied healthcare professionals, administrative staff, etc; students or trainees / staff members)?

________________________________________________________________

Is this support specific to the maternity service or is it available to staff in the maternity service as part of broader hospital/campus staff supports (e.g. available to staff beyond those working in the maternity unit/hospital)?

- Specific to the maternity service
- Available to staff in the maternity service but as part of broader hospital/campus staff supports
- Available to staff in the maternity service but as part of national supports for staff within the health service
- Other, please specify __________________________________________________

How is / can this support be accessed? (E.g. referral / self-referral, automatic referral, etc)

________________________________________________________________

Does access to this support require approval from a person's line manager?

- Yes
- No

How is this support provided: Formally and/or informally? Please tell us as much as you can about how it is provided

________________________________________________________________

What is the format of this support – content, structure, frequency, duration?

________________________________________________________________

How long can this support be accessed for? Are there any limits applied?

________________________________________________________________

Who is this support provided by, and what training/support do they receive (initial/ongoing, including qualifications)?

________________________________________________________________

What costs, if any, are associated with the provision of this support? (e.g. financial, in-kind)

________________________________________________________________

Who covers the costs of providing this support (self- or hospital/HSE-financed)?

________________________________________________________________

Are staff released to attend/avail of this support during work hours or must they do so in their own time (paid/unpaid)?

________________________________________________________________

How are staff made aware that this support is available to them, e.g. how is it advertised and by who?

________________________________________________________________

What is the uptake of this support by staff– by numbers, types of staff? Why / why not? If applicable, have you implemented any strategies to enhance the uptake of supports by staff / certain types of staff?

________________________________________________________________

Has this support/support service been evaluated within your hospital/unit (formally/informally)? If yes, what have the main findings been? Would you be able to share any reports with us?

________________________________________________________________

In your opinion, what has the impact of this support/support service been – e.g. on staff wellbeing and morale, patient care, etc?

________________________________________________________________

Can you share any materials associated with this support/support service with us (e.g. information leaflets, training/procedural manuals, etc)? If yes, please insert links here / email details to maritahennessy@ucc.ie

________________________________________________________________

Why and when was this particular support/support service implemented (e.g. over something else)?

________________________________________________________________

Has anything about this support/support service ever been adapted/anything about it changed? If yes, please specify what and why

________________________________________________________________

End of Block: Block 5_Section C: Staff supports

Start of Block: Block 5_Section C: Staff supports_After Action Rev

**After action review**: A structured review process which seeks to rapidly identify and reinvest learning for improvement

- Yes
- No

Display the same QuestiThis Question:

If After action review: A structured review process which seeks to rapidly identify and reinvest lea... = Yes

[Display the same list of questions as appeared under ‘After Action Reflection’]

End of Block: Block 5_Section C: Staff supports_After Action Rev

Start of Block: Block 5_Section C: Staff supports_Ballint

**Ballint group**: Purposeful, regular meeting among clinicians, facilitated by trained leaders, who discuss the doctor-patient relationship and provide peer support

- Yes
- No

Display This Question:

If Ballint group: Purposeful, regular meeting among clinicians, facilitated by trained leaders, who... = Yes

[Display the same list of questions as appeared under ‘After Action Reflection’]

End of Block: Block 5_Section C: Staff supports_Ballint

Start of Block: Block 5_Section C: Staff Supports_Clin Supv

**Clinical supervision**: Professional relationship between a supervisor and a supervisee where the supervisor facilitates the practitioner in reflecting critically upon their practice (One-to-One)

- Yes
- No

Display This Question:

If Clinical supervision: Professional relationship between a supervisor and a supervisee where the s... = Yes

[Display the same list of questions as appeared under ‘After Action Reflection’]

End of Block: Block 5_Section C: Staff Supports_Clin Supv

Start of Block: Block 5_Section C: Staff supports_Crisis Interv (Other)

**Crisis intervention (other).** Note: Please do not include CISM or TRiM here; they are covered under separate categories

- Yes
- No

Display This Question:

If Crisis intervention (other). Note: Please do not include CISM or TRiM here; they are covered unde... = Yes

[Display the same list of questions as appeared under ‘After Action Reflection’]

End of Block: Block 5_Section C: Staff supports_Crisis Interv (Other)

Start of Block: Block 5_Section C: Staff Supports_Crit Inc Debrief

**Critical incident debriefing** (excluding CISM/TRiM)

- Yes
- No

Display This Question:

If Critical incident debriefing (excluding CISM/TRiM) = Yes

[Display the same list of questions as appeared under ‘After Action Reflection’]

End of Block: Block 5_Section C: Staff Supports_Crit Inc Debrief

Start of Block: Block 5_Section C: Staff Supports_CISM

**Critical Incident Stress Management (CISM) Response**: An emergency mental health intervention, which is a form of psychological first aid

- Yes
- No

Display This Question:

If Critical Incident Stress Management (CISM) Response: An emergency mental health intervention, whi... = Yes

[Display the same list of questions as appeared under ‘After Action Reflection’]

End of Block: Block 5_Section C: Staff Supports_CISM

Start of Block: Block 5_Section C: Staff Supports_EAP

**Employee Assistance Programme (EAP)**: Staff counselling (One-to-One)

- Yes
- No

Display This Question:

If Employee Assistance Programme (EAP): Staff counselling (One-to-One) = Yes

[Display the same list of questions as appeared under ‘After Action Reflection’]

End of Block: Block 5_Section C: Staff Supports_EAP

Start of Block: Block 5_Section C: Staff supports_Hosp psych

**Hospital Psychologist**: Who can provide individual/group counselling and psychological support(s)

- Yes
- No

Display This Question:

If Hospital Psychologist: Who can provide individual/group counselling and psychological support(s) = Yes

[Display the same list of questions as appeared under ‘After Action Reflection’]

End of Block: Block 5_Section C: Staff supports_Hosp psych

Start of Block: Block 5_Section C: Staff supports_Occ health

**Occupational Health: HSE staff**

- Yes
- No

Display This Question:

If Occupational Health: HSE staff = Yes

[Display the same list of questions as appeared under ‘After Action Reflection’]

End of Block: Block 5_Section C: Staff supports_Occ health

Start of Block: Block 5_Section C: Staff supports_Prof (ext) couns

**Professional (external) counselling services (One-to-One)**

- Yes
- No

Display This Question:

If Professional (external) counselling services (One-to-One) = Yes

[Display the same list of questions as appeared under ‘After Action Reflection’]

End of Block: Block 5_Section C: Staff supports_Prof (ext) couns

Start of Block: Block 5_Section C: Staff supports_Schwartz

**Schwartz rounds**: Structured forum where all staff come together regularly to discuss the emotional and social aspects of working in healthcare

- Yes
- No

Display This Question:

If Schwartz rounds: Structured forum where all staff come together regularly to discuss the emotiona... = Yes

[Display the same list of questions as appeared under ‘After Action Reflection’]

End of Block: Block 5_Section C: Staff supports_Schwartz

Start of Block: Block 5_Section C: Staff supports_TRIM

**Trauma Risk Management (TRiM)**: a trauma-focused peer support system designed to help people who have experienced a traumatic, or potentially traumatic, event

- Yes
- No

Display This Question:

If Trauma Risk Management (TRiM): a trauma-focused peer support system designed to help people who h... = Yes

[Display the same list of questions as appeared under ‘After Action Reflection’]

End of Block: Block 5_Section C: Staff supports_TRIM

Start of Block: Block 5_Section C: Staff supports_Add 1

**Additional staff support/support service #1 | Do you wish to provide details about a staff support/support service available to staff in your maternity unit/hospital, which has not been listed already?**

- Yes
- No

Display This Question:

If Additional staff support/support service #1 Do you wish to provide details about a staff support/... = Yes

[Display the same list of questions as appeared under ‘After Action Reflection’]

End of Block: Block 5_Section C: Staff supports_Add1

Start of Block: Block 5_Section C: Staff supports_Add 2

**Additional staff support/support service #2 | Do you wish to provide details about a staff support/support service available to staff in your maternity unit/hospital, which has not been listed already?**

- Yes
- No

Display This Question:

If Additional staff support/support service #2 Do you wish to provide details about a staff support/... = Yes

[Display the same list of questions as appeared under ‘After Action Reflection’]

End of Block: Block 5_Section C: Staff supports_Add 2

Start of Block: Block 5_Section C: Staff supports_Add 3

Display This Question:

If Additional staff support/support service #2 Do you wish to provide details about a staff support/... = Yes

**Additional staff support/support service #3 | Do you wish to provide details about a staff support/support service available to staff in your maternity unit/hospital, which has not been listed already?**

- Yes
- No

Display This Question:

If Additional staff support/support service #3 Do you wish to provide details about a staff support/... = Yes

[Display the same list of questions as appeared under ‘After Action Reflection’]

End of Block: Block 5_Section C: Staff supports_Add 3

Start of Block: Block 6_Section D: Additional comments

**SECTION D: Any other comments**

If there is anything else you would like to tell us about staff education and training around bereavement care, please note it here:

________________________________________________________________

________________________________________________________________

________________________________________________________________

________________________________________________________________

________________________________________________________________

If there is anything else you would like to tell us about staff supports following adverse events, please note it here:

________________________________________________________________

________________________________________________________________

________________________________________________________________

________________________________________________________________

________________________________________________________________

End of Block: Block 6_Section D: Additional comments

Start of Block: Block 7_Thank you

**Thank you for taking the time to complete this survey**
 
**If you have any questions about this study** please contact Marita Hennessy (maritahennessy@ucc.ie) or Professor Keelin O'Donoghue (k.odonoghue@ucc.ie).

End of Block: Block 7_Thank you
