# Supplementary material for: Psychosocial supports for staff in maternity hospitals and units following adverse events: a mapping study in the Republic of Ireland
Source: BMC Health Serv Res. 2026 Mar 30;26:672. doi: 10.1186/s12913-026-14465-7 (PMC13159316; doi:10.1186/s12913-026-14465-7)
Supplement: Supplementary file 2 — Supplementary Material 2 [file 12913_2026_14465_MOESM2_ESM.docx]

**Additional File 3. Additional information – maternity specific or broader, formal/informal support, referral/self-referral, line manager approval,** **released to attend, advertising**

| **Support name** | **Maternity-specific or available to maternity staff as part of national supports for health service staff?** | **Support provided formally or informally?** | **Referral/Self-referral** | **Line manager approval required?** | **How staff facilitated to attend** | **Advertising** |
| --- | --- | --- | --- | --- | --- | --- |
| **Category A: After Action Reflections, After Action Reviews and Critical Incident Debriefing** | | | | | | |
| After Action Reflection (N=5/6) | - Specific to the maternity service (n=1) [S7] - Available to staff in the maternity service but as part of national supports for staff within the health service (n=3) [S3, S15, S18] - “other - maternity, gynae and neonatal” (n=1) [S6] | - Formally (n=2) [S6, S15] - Informally (n=1) [S18] - Both formally and informally (n=2) [S3, S7] | - Self-referral (n=2) [S3, S15] - Referral and self-referral (n=1) [S7] - Unclear (n=2) (“invite issued to staff once date/time of debrief/support session is arranged” [S18]; “quality manager, Master/CEO, Director of Midwifery” [S6]); neither specified if this referral was automatic or on request | - No (n=3) [S3, S7, S18] - Yes (n=2) [S6, S15] | - Paid to attend (n=2) [S3, S6] - Usually released during working hours (n=1) [S7] - Depends – if on duty released or paid if availed of outside of rostered hours (n=2) [S15, S18] | - Targeted, e.g. via line mangers / (A)DOM following event (n=4) [S6, S7, S15, S18] - Advertising locally, e.g. on wards (n=2) [S3, S18] - Staff policies (n=1) [S3] |
| After Action Review (N=7/9) | - Specific to the maternity service (n=4) [S5, S7, S8, S15] - Available to staff in the maternity service but as part of broader hospital/campus staff supports (n=2) [S3, S16] - Both (n=1) [S10] | - Formally (n=7) [S3, S5, S7, S8, S10, S15, S16] | - Automatic referral (n=2) [S3, S7] - Automatic / self-referral (n=1) [S5, S10] - “Those involved will be asked” (n=1) [S15] - Unclear (n=2) (“Email, phone or word of mouth” [S8]; “Through Clinical risk manager” [S16]) | - No (n=6) [S3, S5, S7, S10, S15, S16] - Yes (n=1) [S8] | - Released to attend during work hours (n=4) [S3, S7, S8, S10] - Released if on duty or can attend on their own time and get time in lieu (n=2) [S5, S16] with a further site implying this but not specifying if staff would receive time in lieu for attending out of hours [S15] (n=1) | - Line manager / management (n=4) [S5, S10, S15, S16] - Email (n=2) [S5, S10] - Safety pause / education sessions (n=2) [S5, S8] - Posters (n=2) [S5, S8] - Leaflets (n=1) [S8] - Unclear (n=2) (“made aware locally” [S3]; “individuals” [S10]) - Not stated (n=1) [S7] |
| Critical Incident Debriefing (N=7/9) | - Specific to the maternity service (n=3) [S5, S11, S13] - Available to staff in the maternity service but as part of broader hospital/campus staff supports (n=4) [S3, S4, S9, S18] | - Formally (n=1) [S3] - Formally and informally (n=2) (“by CSF and consultants” / “both (formally and informally)” [S4]; “Support ? Varies and no consistent approach. Can be informal depending on facilitator - and is group based” [S11]) - “informally through line manager” (n=1) [S5] - Unclear (n=2) (“Through EAP” [S9]; “Facilitated by QPS department” [S18].) - Don’t know (n=1) [S13] | - Automatically (n=2) [S3, S9] - Self-referral (n=1) [S5] - Unclear (n=3) (“through their CMM2 or CSF” / “Risk management meetings” [S4]; “face to face” [S13]; “Locally, invite with date/time/venue.” [S18]) - S11 noted “not accessed - decision by management to hold a debriefing - but no consistent approach to offering / running same” (n=1) | - No (n=5) [S3, S4, S5, S11, S13] - Yes (n=2) [S9, S18] | - Released to attend during work hours (n=5) [S3, S4, S5, S9, S18] - “[it] Varies and people attend in their own time” (n=1) [S11] - Unsure (n=1) [S13] | - Via line/QPS manager (n=3) [S5, S9, S18] - “spoken with after the event, posters in tearoom and newsletter” / “emails” (n=1) [S4] - Unclear: “on being established” (n=1) [S13] - S11 highlighted that staff were “Not always” made aware that this support is available to them (n=1) - No response (n=1) [S3] |
| **Category B: Critical Incident Stress Management (CISM) and Trauma Risk Management (TRiM)** | | | | | | |
| CISM (N=4/5) | - Specific to the maternity service (which it is not) (n=1) [S10] - Available to staff in the maternity service but as part of national supports for staff within the health service (n=3) [S4, S7, S9] | - Formally (n=4) [S4, S7, S9, S10] – including provided through EAP/CISM Officer [S4, S9] | - Referral (n=1) [S10] - Self-referral (n=1) [S7] - Referral and self-referral (n=2) [S4] - “Offered to staff involved in the incident” (n=1) [S9] | - No (n=2) [S4, S7] - Yes (n=2) [S9, S10] | - Released to attend during work hours (n=3) [S4, S9, S10] - Released to attend or can do in their own time (n=1) [S7] | - Via line manager [S7, S9, S10] - Via “CMM3, DOM or CSF” (n=1) [S4] - Occupational health (n=1) [S7] - Posters in tearoom (n=1) [S4] |
| TRIM (N=4/4) | - Specific to the maternity service (n=4) [S8, S10, S11, S13] | - Formally (n=4) [S8, S10, S11, S13] | - Self-referral or referral (n=3) [S8, S10, S11] including via a person’s line manager [S10] - Automatic (n=2) [S10, S13] | - No (n=4) [S8, S10, S11, S13] | - Released to attend during work hours (n=2) [S8, S10] - Attend mostly in own time (n=1) [S11] - “flexible” (n=1) [S13] | - Emails (n=1) [S10] - Word of mouth (n=1) [S10] - Posters (n=1) [S8] - Education sessions/clinical meetings (n=2) [S8, S13] - Line manager (n=1) [S10] - Unclear (n=1) (“widely advertised as a pilot from December 23” [S11]) |
| **Category C: Schwartz Rounds** | | | | | | |
| Schwartz Rounds (N=3/5) | - Specific to the maternity service (n=1) [S17] - Available to staff in the maternity service but as part of broader hospital/campus staff supports (n=1) [S5] - “maternity, gynae, neonatal” only (n=1) [S6] | - Formally (n=2) [S5, S6] - Informally (n=1) [S17] | - Self-referral (n=1) [S6] - Unclear (“Open to everyone” [S5]; “advertised via posters/email” [S17]) | - No (n=3) [S5, S6, S17] | - Released to attend during work hours (n=1) [S5] - Paid (n=1) [S6] - Not stated (n=1) [S17] | - Posters (n=3) [S5, S6, S17] - Email (n=2) [S5, S17] - Intranet (n=1) [S6] - Safety pauses (n=1) [S5] - Line managers (n=1) [S5] |
| **Category D: Clinical Supervision, Employee Assistance Programme: Staff Counselling (One-to-One), Hospital Psychologist, Occupational Health, Professional (External) Counselling Services (One-to-One)** | | | | | | |
| Clinical Supervision (N=10) | - Specific to the maternity service (n=4) [S3, S11, S12, S18] - Available to staff in the maternity service but as part of national supports for staff within the health service (n=4) [S4, S7, S9, S10] - Other: *“maternity, gynae and neonatal”* (n=1) [S6] - Unclear (n=1) (“CMS only” [S13]) | - Formally (n=8) [S3, S4, S6, S9, S10, S11, S13, S18] - Formally and informally (n=1) (*“formally (for CMS bereavement), informally for peer supervision/support”* [S7]) - Not specified (n=1) [S12] | - Referral (n=1) [S10] - Self-referral (n=5) [S3, S4, S6, S9, S11] - Privately accessed but funded by hospital (n=1) [S12] - Request (n=1) [S18] - Not specified (n=2) [S7, S13] | - No (n=1) [S7] - Yes (n=9) [S3, S4, S6, S9, S10, S11, S12, S13, S18] | - Released to attend during work hours (n=6) [S3, S7, S9, S10, S13, S18] - Attend in own time (n=1) [S11] - Released to attend during work hours or attend in own time (n=1) [S4] - Unclear (n=1) (“paid” [S6] - No response (n=1) [S12] | - Line manager (n=3) [S6, S7, S9] - Job description (n=2) [S3, S10] - Via another CMS-BL (n=2) [S4, S18] - Not applicable (n=1) [S13] (only applicable to them as a CMS-BL) - Not specified (n=1) (“only those to whom it applies - limited” [S11]) - No response (n=1) [S12] |
| EAP: Staff Counselling (N=15/16) | - Available to staff in the maternity service as part of broader hospital/campus staff supports (n=14) [S1, S2, S3, S4, S5, S7, S9, S10, S11, S12, S13, S15, S17, S18] - “Other - maternity, gynae and neonatal” (n=1) [S6] | - Formally (n=8) [S1, S4, S5, S7, S9, S10, S11, S15] - Informally (n=1) [S18] - Formally and informally (n=2) [S6, S12] - Not specified (n=3), but noted format (“Through counselling sessions” [S2] and “phone consultation & face to face” [S13]) or how staff were made aware of EAP (“information on EAP is in poster form around hospitals; and managers are aware of this support” [S17]) - No response (n=1) [S3] | - Sites noted that EAP access was via self-referral (n=10) [S1, S2, S3, S6, S9, S10, S12, S15, S17, S18] - Referral/self-referral (n=5) [S4, S5, S7, S11, S13] - S18 alluded to the individual and group offerings available via EAP: “Self referral. Also, in event of SAE, line manager will co-ordinate group EAP debrief on site”. | - No (n=15) [S1, S2, S3, S4, S5, S6, S7, S9, S10, S11, S12, S13, S15, S17, S18] | - Attend in own time and/or are not paid to attend (n=7) [S1, S4, S6, S10, S11, S15, S17] - Released to attend during work hours (n=2) [S5, S9] - Released and/or can attend in own time (n=4) [S2, S7, S13, S18], with S18 noting, “if off duty, they can claim for pay” - No response (n=2) [S3, S12] | - Posters/flyers within the hospital – noticeboard, tearoom (n=8) [S1, S2, S4, S5, S7, S9, S13, S17] - Online/HSE website (n=4) [S1, S9, S10, S11] - Email (n=5) [S1, S4, S5, S11, S13] - Safety pause / staff meetings (n=2) [S5, S18] - Study days (n=1) [S6] - Induction/orientation programmes (n=3) [S6, S11, S18] - Line manager (n=5) [S6, S7, S10, S11, S17] - Human resources (n=1) [S6] - Occupational health (n=1) [S7] - Contact details on ID cards (n=1) [S15] - Unclear (n=2) (“in aftermath of SAE” [S18]; “advertised throughout hospital” [S15])” - No response (n=2) [S3, S12] |
| Hospital Psychologist (N=3/5) | - Specific to the maternity service (n=2) [S10, S13] - Available to staff in the maternity service but as part of broader hospital/campus staff supports (n=1) [S7] | - Formally (n=3) [S7, S10, S13] | - Self-referral (n=1) [S13] - “Self referral or referral by Line Manager” (n=1) [S10] - “referral via occ health” (n=1) [S7] | - No (n=3) [S7, S10, S13] | - Released to attend during work hours or in own time (n=1) [S7] - Released during work hours (n=1) [S10] - Unsure (n=1) [S13] | - Line manager (n=2) [S7, S10] - Occupational Health (n=1) [S7] - Word of mouth (n=1) [S13] - Psychologist visits the maternity unit (n=1) [S13] |
| Occupational Health (N=14/15) | - Specific to the maternity service (n=1) [S17] - Available to staff in the maternity service but as part of broader hospital/campus staff supports (n=12) [S1, S2, S3, S4, S5, S7, S10, S11, S12, S15, S16, S18] - “Other - maternity, gynae and neonatal” (n=1) [S6] | - Formally (n=9) [S1, S4, S5, S6, S7, S10, S11, S15, S16] - Formally and informally (n=1) [S12] - Unsure (n=2) [S17, S18] - Not stated (n=2) [S2, S3] | - Self-referral / referral (n=6) [S2, S6, S7, S10, S12, S17] - Referral (n=5) [S4, S11, S15, S16, S18] - Self-referral (n=1) [S1] - Referral / self-referral / automatic referral (n=1) [S5] - No response (n=1) [S3] | - No (n=9) [S1, S4, S5, S6, S7, S10, S12, S16, S17] - Yes (n=4) [S2, S11, S15, S18] - No response (n=1) [S3] | - Access in own time (whether paid/not, not specified) (n=3) [S4, S11, S15] - Released to attend during work hours (n=2) [S2, S5] - Paid (not specified if released or on own time) (n=1) [S6] - Released or attend in own time (n=3) [S7, S10, S18], with S18 noting that “If off duty, can claim pay” and S10 noting that “[staff are] released if referred by Line Manager/ own time if a personal referral” - S16 stated “Yes [own time] unpaid”. (n=1) - Unsure (n=1) [S17] - No response (n=3) [S1, S3, S12] | - Line managers (n=9) [S4, S5, S6, S7, S10, S11, S15, S16, S18] - Recruitment/orientation/induction programmes (n=4) [S6, S7, S11, S18] - Online/HSE website (n=2) [S5, S11] - Email (n=1) [S5] - Posters (n=1) [S5] - Safety pause/staff meetings (n=2) [S5, S18] - GP (n=1) [S4] - “not really [advertised]” (n=1) [S17] - No response (n=4) [S1, S2, S3, S12] |
| Professional Counselling (N=4/5) | - Specific to the maternity service (n=3) [S5, S16, S18] - Available to staff in the maternity service as part of broader hospital/campus staff supports (n=1) [S17] | - Formally (n=3) [S5, S16, S18], with S18 further noting that *“Staff member sources psychologist and submits invoice for payment to line manager”* - No response (n=1) [S17] | - Self-referral (n=2) [S5, S17] - Following “Discussion with line manager / DOM. Approval by GM” (n=1) [S18] - Not stated (n=1) [S16] – but noted that “Suitable counsellor identified to support CMSp generally on recommendation” | - No (n=2) i.e. those with self-referral processes [S5, S17] - Yes (n=2) [S18, S16] | - Staff released to attend during work hours (n=2) [S5, S16] - Could be outside of work hours (n=2) [S17, S18] | - Line manager(s) (n=3) [S5, S16, S17] - Posters (n=1) [S17] - “not advertised. Not widely available, specific circumstances e.g. following inquest.” [S18] |

CEO: Chief Executive Officer, CMM: Clinical Midwife Manager, CMS: Clinical Midwife Specialist, CMS-BL: Clinical Midwife Specialist in Bereavement and Loss, CSF: Clinical Skills Facilitator, (A)DOM: (Assistant)Director of Midwifery, EAP: Employee Assistance Programme, GM: General Manager, GP: General Practitioner, HSE: Health Service Executive, QPS: Quality and Patient Safety, SAE: Serious Adverse Event, S: Site.
