# Supplementary material for: Psychosocial supports for staff in maternity hospitals and units following adverse events: a mapping study in the Republic of Ireland
Source: BMC Health Serv Res. 2026 Mar 30;26:672. doi: 10.1186/s12913-026-14465-7 (PMC13159316; doi:10.1186/s12913-026-14465-7)
Supplement: Supplementary file 3 — Supplementary Material 3 [file 12913_2026_14465_MOESM3_ESM.docx]

**Additional File 4. Additional information – materials, costs, reasons for introduction, adaptations**

| **Support name** | **Availability of materials** | **Costs (what & who covers)** | **Reason(s) for introduction** | **Any adaptations made** |
| --- | --- | --- | --- | --- |
| **Category A: After Action Reflections, After Action Reviews and Critical Incident Debriefing** | | | | |
| After Action Reflection (N=5/6) | - Cannot be shared; no materials used or not applicable often stated (n=4) [S3, S6, S7, S15] - “EAP” (n=1) [S18] | *Costs*   - No associated costs (n=5) [S3, S6, S7, S15, S18]   *Who covers costs*   - HSE/hospital (n=3) [S3, S6, S18], S6 and S18 noted costs in the form of staff pay - Not applicable (n=2) [S7, S15] | - Important, evidence-based, effective support mechanism (n=2) [S7, S18] - Following an adverse event (maternal death)/the need for staff supports (n=1) [S6] - National guidance (n=1) [S15] - No response (n=1) [S3] | - None (to their knowledge) (n=2) [S15, S18] - No response (n=3) [S3, S6, S7] |
| After Action Review (N=7/9) | - “all on HSE” (n=1) [P5] - “Example - provided on FirstLight website” / “national template” (n=1) [P10] - No (n=2) [P3, P15] - No response (n=3) [P7, P8, P16] | *Costs*   - No associated costs (n=6) [S3, S5, S7, S8, S15, S16] - “Financial” / “HSE financed” costs (n=1) [S10]   *Who covers costs*   - HSE/Hospital (n=3) [S3, S5, S10] - Not applicable (n=4) [S7, S8, S15, S16] | - Based on need [S8, S10] and/or in line with (national) policy/framework [S10, S15, S16] (n=5) - Year of introduction noted (2018) (n=1) [S5] - No response (n=2) [S3, S7] | - None (n=3) [S5, S15, S16] - “Under national advice only” (n=1) [S10] - No response (n=3) [S3, S7, S8] |
| Critical Incident Debriefing (N=7/9) | - *“*assist me model, HSE” (n=1) [S5] - No / not available (n=3) [S4, S9, S18] - Don’t know (n=1) [S13] - No response (n=2) [S3, S11] | *Costs*   - No associated costs (n=6) [S3, S4, S5, S11, S18] - Do not know (n=2) [S9, S13]   *Who covers costs*   - HSE/Hospital (n=3) [S4, S9, S18] - Don’t know (n=1) [S13] - Not applicable (n=1) [S5] - No response (n=2) [S3, S11] | - Improve care quality and/or help staff (n=2) (“to help staff process traumatic incidents at work” / “To improve quality of care” [S4] and “initiated by QPS dept as part of IMF recommendations” [S18]) - Don’t know (n=2) [S9, S13] - No response (n=3) [S3, S5, S11] | - Yes, *“*adapted every meeting depending on the case and disciplines involved” / “Yes, changes in practice” (n=1) [S4] - “No - needs to be formalized” (n=1) [S11] - No – only introduced (n=1) [S13] - Do not know (n=2) [S9, S18] - No response (n=2) [S3, S5] |
| **Category B: Critical Incident Stress Management (CISM) and Trauma Risk Management (TRiM)** | | | | |
| CISM (N=4/5) | - No (n=4) [S4, S7, S9, S10]; S4 highlighted *“all used by EAP or CISM officers”* | *Costs*   - “Financial” (n=1) [S10] - None (n=1) [S7] - Unknown (n=2) [S4, S9]   *Who covers costs*   - HSE/Hospital (n=4) [S4, S7, S9, S10] | - In response to particular traumatic/serious incident [S4, S10] or need [S7] (n=3) - “not known” (n=1) [S9] | - “more awareness and availably” (n=1) [S7] - No (n=1) [S4] - Don’t know (n=1) [S9] - No response (n=1) [S10] |
| TRIM (N=4/4) | - Yes (n=2) [S10, S11], with S11 specifying *“TRIM leaflet”* - No (n=2) [S8, S13] | *Costs*   - No costs to staff (n=3) [S8, S10, S11] - No response (n=1) [S13]   *Who covers costs*   - Hospital group (n=3) [S10, S11, S13] - Not applicable (n=1) [S8] | - Need to better support staff following potentially traumatic events (n=3) [S8, S10, S13] - Championed by the staff psychologist (n=1) [S11] | - No (n=1) [S8] - Not applicable – only recently available to staff (n=2) [S10, S11] - No response (n=1) [S13] |
| **Category C: Schwartz Rounds** | | | | |
| Schwartz Rounds (N=3/5) | - No (n=1) [S6] - No response (n=2) [S5, S17] | *Costs*   - “financial” (n=1) [S6] - “none” (n=1) [S5] - No response (n=1) [S17]   *Who covers costs*   - Hospital (n=2) [S5, S6] - No response (n=1) [S17] | - [Introduced in] 2021 (n=1) [S6] - Unsure (n=1) [S17] - No response (n=1) [S5] | - No (n=1) [S6] - Unsure (n=1) [S17] - No response (n=1) [S5] |
| **Category D: Clinical Supervision, Employee Assistance Programme: Staff Counselling (One-to-One), Hospital Psychologist, Occupational Health, Professional (External) Counselling Services (One-to-One)** | | | | |
| Clinical Supervision (N=10) | - No/not available (n=7) [S3, S4, S6, S9, S10, S11, S18] - No response (n=3) [S7, S12, S13] | *Costs*   - Financial (n=3) [S3, S6, S10], with S3 noting “monthly cost of 140 euro” - HSE/Hospital (n=3) [S4, S11, S18] - Paid by employer (n=1) [S9] - Funded (n=1) [S12] - None (n=1) [S13] - Don’t know (n=1) [S7]   *Who covers costs*   - HSE/Hospital (n=10) [S3, S4, S6, S7, S9, S10, S11, S12, S13, S18] | - Year of introduction reported, which many noted coincided with when CMS-BL post was introduced or commenced (n=6) [S4, S6, S10, S11, S13, S18] - Don’t know (n=1) [S9] - No response (n=3) [S3, S7, S12] | - No (n=3) [S6, S10, S11], with S11 noting that it’s “still limited to a few staff members” - “Yes, coping skills” [S4] (n=1) - “between myself & the psychologist we discuss what works & what doesn't” (n=1) [S13] - Don’t know or unsure (n=2) [S9, S18] - No response (n=3) [S3, S7, S12] |
| EAP: Staff Counselling (N=15/16) | - No (n=7) [S4, S6, S7, S9, S15, S17, S18], with one noting that these took the form of information leaflets [S4] - Can be accessed on the HSE website (n=2) [S10, S11] - No response (n=6) [S1, S2, S3, S5, S12, S13] | *Costs*   - No costs/free (n=10) [S1, S2, S5, S7, S9, S10, S11, S13, S15, S18] - “financial” costs (n=1) [S6] - “funded” (n=1) [S12] - Do not know (n=2) [S4, S17] - No response (n=1) [S3]   *Who covers costs*   - HSE/Hospital (n=13) [S1, S2, S4, S5, S6, S7, S9, S10, S11, S13, S15, S17, S18] - No response (n=2) [S3, S12] | - When introduced (n=5) (“2005/2006” [S6]; “early 2000 – requirement identified” [S7]; “implemented a number of years” [S18]) and/or who introduced it (“HSE” [S9]; “As part of staff occupational health service- on-going for years” [S11]”) - Why introduced/who or what it was for (n=2) (“work related stress” [S4]; “For work based or penal reason for all HSE Staff” [S10]) - Do not know (n=2) [S15, S17] - No response (n=6) [S1, S2, S3, S5, S12, S13] | - “more available and utilized more” (n=1) [S7] - “yes coping skills [Teaches you coping strategies]” (n=1) [S4] - No (n=4) [S6, S9, S15, S17] - Don’t know (n=2) [S11, S18] - No response (n=7) [S1, S2, S3, S5, S10, S12, S13] |
| Hospital Psychologist (N=3/5) | - No (n=1) [S7] - “HSE” (n=1) [S10] - No Response (n=1) [S13] | *Costs*   - No costs (n=2) [S7, S10] - “HSE” (n=1) [S13]   *Who covers costs*   - HSE (n=2) [S7, S13] - “one directorate” (n=1) [S10] | - Need *“identified”* [S7] - Year of introduction (n=2) (*“New service since May 2022”* [S10]; *“2023”* [S13]) | - “Yes – reflections as per staff needs” (n=1) [S10] - No response (n=2) [S7, S13] |
| Occupational Health (N=14/15) | - “HSE website” (n=1) [S11] - No (n=7) [S6, S7, S10, S15, S16, S17, S18] - No response (n=6) [S1, S2, S3, S4, S5, S12] | *Costs*   - No costs (n=8) [S2, S5, S7, S10, S11, S16, S17, S18] - “financial” (n=1) [S6] - Unsure (n=2) [S4, S15] but “no cost to employee” [S15] - No response (n=3) [S1, S3, S12]   *Who covers costs*   - HSE/Hospital (n=11) [S2, S4, S5, S6, S7, S10, S11, S12, S15, S16, S18] - Unsure (n=1) [S17] - No response (n=2) [S1, S3] | - When and/or why introduced (n=4): “2010” [S6]; “HSE” [S10]; “In line with national policy” [S15]; “Part of staff support package for a number of years” [S18] - Unsure (n=3) [S11, S16, S17] - No response (n=7) [S1, S2, S3, S4, S5, S7, S12] | - Nothing (n=1) [S15] - Unclear (n=2) (“increase our service Monday to Friday, 37.5 by CNS” (n=1) [S6]; “health and wellbeing unit, Dublin” [S10]) - Don’t know (n=4) [S11, S16, S17, S18] - No response (n=7) [S1, S2, S3, S4, S5, S7, S12] |
| Professional Counselling (N=4/5) | - No (n=4) [S5, S16, S17, S18] | *Costs*   - *“Per Session”* (n=1) [S5] - *“Financial”* (n=1) [S18] - Don’t know (n=2) [S16, S17]   *Who covers costs*   - HSE/Hospital (n=4) [S5, S16, S17, S18] | - “Part of appointment to post” (n=1) [S16] - “to support staff who feel vulnerable following involvement in SAE and subsequent inquest” (n=1) [S18] - Unsure (n=1) [S17] - No response (n=1) [S5] | - No (n=2) [S16, S18] - Unsure (n=1) [S17] - No response (n=1) [S5] |

CMS-BL: Clinical Midwife Specialist in Bereavement and Loss, CNS: Clinical Nurse Specialist, EAP: Employee Assistance Programme, HSE: Health Service Executive, IMF: Incident Management Framework, QPS: Quality and Patient Safety, S: Site.
