# Supplementary material for: Psychosocial supports for staff in maternity hospitals and units following adverse events: a mapping study in the Republic of Ireland
Source: BMC Health Serv Res. 2026 Mar 30;26:672. doi: 10.1186/s12913-026-14465-7 (PMC13159316; doi:10.1186/s12913-026-14465-7)
Supplement: Supplementary file 4 — Supplementary Material 4 [file 12913_2026_14465_MOESM4_ESM.docx]

**Additional File 2. Details of the 19 maternity hospitals and units by category, hospital group, HSE health region and number of births**

| **Maternity hospital or unit name** | **Maternity unit or hospital category** | **Hospital group**  **(pre-2024)** | **HSE health region (2024-)** | **No. of births, 2022^1^** |
| --- | --- | --- | --- | --- |
| Coombe Hospital | Maternity hospital | Dublin Midlands | Dublin and Midlands | 6,916 |
| Midland Regional Hospital Mullingar | Maternity unit | Ireland East | Dublin and Midlands | 1,732 |
| Midland Regional Hospital Portlaoise | Maternity unit | Dublin Midlands | Dublin and Midlands | 1,362 |
| Cavan Monaghan Hospital | Maternity unit | RCSI | Dublin and North East | 1,257 |
| Our Lady of Lourdes Hospital Drogheda | Maternity unit | RCSI | Dublin and North East | 2,867 |
| Rotunda Hospital | Maternity hospital | RCSI | Dublin and North East | 8,293 |
| National Maternity Hospital, Holles Street | Maternity hospital | Ireland East | Dublin and South East | 6,911 |
| St Luke's General Hospital Kilkenny | Maternity unit | Ireland East | Dublin and South East | 1,398 |
| Tipperary University Hospital | Maternity unit | South South West Hospital Group | Dublin and South East | 796 |
| University Hospital Waterford | Maternity unit | South South West Hospital Group | Dublin and South East | 1,677 |
| Wexford General Hospital | Maternity unit | Ireland East | Dublin and South East | 1,569 |
| University Maternity Hospital Limerick | Maternity hospital | UL Hospitals | Mid West | 3,931 |
| Cork University Maternity Hospital | Maternity hospital | South South West Hospital Group | South West | 6,538 |
| University Hospital Kerry | Maternity unit | South South West Hospital Group | South West | 1,131 |
| Letterkenny University Hospital | Maternity unit | Saolta | West and North West | 1,495 |
| Mayo University Hospital | Maternity unit | Saolta | West and North West | 1,377 |
| Portiuncula University Hospital | Maternity unit | Saolta | West and North West | 1,399 |
| Sligo University Hospital | Maternity unit | Saolta | West and North West | 1,241 |
| University Hospital Galway | Maternity unit | Saolta | West and North West | 2,649 |
| **Total** |  |  |  | **54,539** |

^1^Source: National Women and Infants Health Programme. Annual Report 2022. Dublin: National Women and Infants Health Programme; 2022.
